# An update and revision of the *Andrena* fauna of Morocco (Hymenoptera, Apoidea, Andrenidae) with the description of eleven new North African species

**DOI:** 10.3897/zookeys.974.54794

**Published:** 2020-10-07

**Authors:** Thomas James Wood, Denis Michez, Diego Cejas, Patrick Lhomme, Pierre Rasmont

**Affiliations:** 1 Laboratoire de Zoologie, Université de Mons, 7000, Mons, Belgium Université de Mons Mons Belgium; 2 International Center of Agricultural Research in the Dry Areas, Rabat, Morocco International Center of Agricultural Research in the Dry Areas Rabat Morocco

**Keywords:** alpine, deserts, endemic, faunal list, pollen host plants, solitary bees, taxonomy

## Abstract

Morocco has a diverse bee fauna, but one that has also been relatively understudied in recent years. Here a revision of the species-rich genus *Andrena* is presented that reveals eleven new species for science and substantially improves our understanding of North African *Andrena*. From Morocco, Andrena (Aciandrena) semiadesus Wood, **sp. nov.**, Andrena (Aciandrena) triangulivalvis Wood **sp. nov.**, Andrena (Campylogaster) sparsipunctata Wood **sp. nov.**, Andrena (Carandrena) hebescens Wood **sp. nov.**, Andrena (Cnemidandrena) niveofacies Wood **sp. nov.**, *Andrena* (*incertae sedis*) *tenebricorpus* Wood **sp. nov.**, Andrena (Notandrena) acutidentis Wood **sp. nov.**, Andrena (Poliandrena) breviceps Wood **sp. nov.**, and Andrena (Poliandrena) farinosoides Wood **sp. nov.** are described and their ecology is discussed. Andrena (Aciandrena) astrella Warncke, 1975 is synonymised with Andrena (Aciandrena) fulica Warncke, 1974 **syn. nov.** The unknown female of Andrena (Nobandrena) ounifa Warncke, 1974, and the unknown male of Andrena (Poliandrena) guichardi Warncke, 1980 are described. *Andrena* (*incertae sedis*) *gafsensis* Wood **sp. nov.** from Tunisia is described due to its similarity to *Andrena tenebricorpus*. Andrena (Poecilandrena) nigriclypeus Wood **sp. nov.** from Algeria is also described as it was collected within 10 km of the Moroccan border. A further 18 species are recorded in Morocco for the first time. Andrena (Melandrena) nitida (Müller, 1776) and Andrena (Notandrena) nitidiuscula Schenck, 1853 are removed from the Moroccan list due to historic problems in the application of these names to Mediterranean taxa.

## Introduction

*Andrena* are the second most speciose genus of bees worldwide after *Lasioglossum* (Ascher and Pickering 2020). The genus has a primarily Holarctic distribution and with the greatest diversity found in Mediterranean and xeric regions (Gusenleitner and Schwarz 2002). The Mediterranean basin of the Western Palearctic is therefore one of the hotspots of *Andrena* diversity, and new *Andrena* species continue to be described from North Africa and the Levant at regular intervals (e.g., Scheuchl 2009; Scheuchl and Gusenleitner 2009; Scheuchl et al. 2011; Schwenninger 2015; Pisanty et al. 2016; Pisanty et al. 2018). Compared to the Levant, the *Andrena* fauna of north-western Africa is historically better known and described thanks to a long history of taxonomic activity (e.g., Pérez 1895; Schmiedeknecht 1900; Saunders 1908; Alfken 1914; Schulthess 1924; Benoist 1961). The *Andrena* fauna of this region was later revised by Warncke (1967, 1974, 1980, 1983) who described many new species. Since then there has been relatively little work conducted on North African *Andrena* until recently (Scheuchl 2009; Scheuchl et al. 2011; Benarfa et al. 2013; Cherair et al. 2013; Schwenninger 2015; Djouama et al. 2016) as interest in the conservation, ecology, and taxonomy of wild bees has increased. However, nothing on the taxonomy of *Andrena* in Morocco has been published since 1980 (Warncke 1980), and a critical reappraisal of this rich fauna is therefore warranted.

Inspection of 5,685 female and 2,275 male *Andrena* specimens from contemporary and ongoing collections in Morocco and undetermined material from the Oberösterreich Landesmusum, Linz, the Naturalis Biodiversity Center, Leiden, and the personal collection of Maximillian Schwarz, Ansfelden, comprised 155 valid species, with many new records for Morocco and undescribed taxa. These are described herein, and the identity of several problematic taxa are reviewed.

## Materials and methods

Identification was enabled through a comprehensive review of the literature in combination with visits to the Warncke collection in Linz as part of the lead author’s ongoing review of West Palearctic *Andrena* species (Wood 2020; Wood et al. 2020a, b).

The subgeneric classification of *Andrena* continues to pose problems. Because of the very large number of species, taxonomic workers have largely been restricted to only a part of the global fauna (broadly, West Palearctic, Central and Eastern Palearctic, and Nearctic falling into three groups), and therefore a co-ordinated global system of subgeneric classification has not yet been possible. In both a Moroccan and West Palearctic context, the system of Warncke (1968a) is the most relevant, and so we broadly follow his assigned subgenera. However, though no globally consistent system currently exists, major steps towards such a reclassification have recently been made (Pisanty et al. 2020). Molecular analyses show that many existing *Andrena* subgenera are strongly polyphyletic (particularly groups such as *Poecilandrena* and *Poliandrena*), and are in need of deep revision. It is currently beyond the scope of this study to revise the subgeneric status of Moroccan *Andrena* taxa, but we take the opportunity to discuss problematic taxa, highlight outstanding areas of confusion, and to place newly described taxa with an appropriate degree of confidence in light of the findings of Pisanty et al. (2020).

Body length was measured from the vertical plane of the front of the head to the tip of the metasoma. Morphological terminology follows Michener (2007). Photographs were taken using an Olympus E-M1 Mark II with a 60 mm macro lens and were stacked using Zerene Stacker 1.04 (Zerene Systems, USA) and plates were prepared in GNU Image Manipulation Program (GIMP) 2.10.

In order to investigate a possible synonymy (see section on *Andrena alchata* Warncke, 1974), DNA was extracted and amplified from two specimens. DNA was extracted from two legs of each individual using a NucleoSpin Tissue (Macherey-Nagel, Düren Germany) extraction kit and following manufacturer´s instructions. The region selected for amplification was the LEP fragment within the mitochondrial gene cytochrome oxidase I (*cox1*), a region widely used in Hymenoptera taxonomy (Sheffield et al. 2009). However, as retrieving genetic information from pinned specimens can be challenging (Wandeler et al. 2007), a section within the fragment was targeted for amplification using primers LEP-F1 and LEP-R2 (Hebert et al. 2004). PCR profile consisted of an initial denaturation step at 94 °C for 3 min followed by 36 cycles of denaturation at 94 °C for 1 min, annealing temperature at 50 °C for 1 min and elongation at 72 °C for 1 min followed by a final extension at 72 °C for 10 min. Results of the PCR reaction were checked in a 1.5% agarose gel. Amplicons were sequenced using forward and reverse primers (Eurofins, Germany).

**MSC** Maximillian Schwarz personal collection, Ansfelden, Linz, Austria

**NMNL** National Museum of Natural History Naturalis, Leiden, the Netherlands

**OÖLM** Oberösterreich Landesmusum, Linz, Austria

**TJW** Thomas Wood personal collection, Mons, Belgium

**UMONS** University of Mons collection, Mons, Belgium

## Results

### Description of new species

#### Andrena (Aciandrena) semiadesus

Taxon classificationAnimaliaHymenopteraAndrenidae

Wood sp. nov.

E9BC8D33-FE6E-54EF-8B35-0FE4F44EBDB0

http://zoobank.org/235512E8-91EA-4A84-91B8-41F7727AE452

Figures 1–8

##### Material.

***Holotype***: Morocco: Fès-Meknès, Laanoucer, 1456 m, 33.6166N, -4.7484W, 11–12.iv.2019, 1♂, white pan trap, leg. L. Hamroud & A. Sentil. Deposited in the OÖLM. ***Paratypes***: Morocco: Fès-Meknès, Laanoucer, 1456 m, 11–12.iv.2019, 4♂, white and yellow pan traps, leg. L. Hamroud & A. Sentil; Drâa-Tafilalet, 20 km W Boudnib, 9.iv.1995, 2♂, 2♀, leg. Ma. Halada. Paratypes are deposited at the OÖLM, with a male and female retained in the personal collection of TJW.

##### Diagnosis.

The finely shagreened propodeal triangle, the narrow facial foveae, the absence of longitudinal striations on the clypeus, and the yellow-marked clypeus in the male place this bee in the *Aciandrena*. The classification of *Aciandrena* and its relationship to *Micrandrena*, *Graecandrena*, and Distandrena is somewhat in flux, and the subgenus is currently polyphyletic (Pisanty et al. 2020). However, when excluding uncharacteristic taxa that are currently placed in the *Aciandrena* such as *A. janthina* Warncke, 1975 that clearly belong elsewhere (Pisanty et al. 2020), *A. semiadesus* meets the classical definition of *Aciandrena*. The male can be instantly separated from the other *Aciandrena* with yellow clypei because the yellow marking is diminished, it does not extend all the way to the clypeal margins, and the two dark spots that are usually found towards the centre of the clypeus in yellow-faced *Aciandrena* are absent as they instead are contiguous with black markings that extend in from the clypeal margins (Fig. 6). The genitalia are also noticeably more elongate, the capsule almost twice as long as wide, and with a broad penis valve, most other *Aciandrena* species with capsules only a little longer than wide and with a comparatively narrow penis valve (Figs 100, 102, 104, 126, 128, 130; Pisanty et al. 2016). The female is most similar to A. (Graecandrena) totana Warncke, 1974 as they both have a broad, shagreened, dull, moderately raised, and centrally slightly flattened sparsely punctate clypeus, but the tergites of *A. semiadesus* are completely impunctate and the foveae are narrower.

##### Description.

**Figures 1–8.**
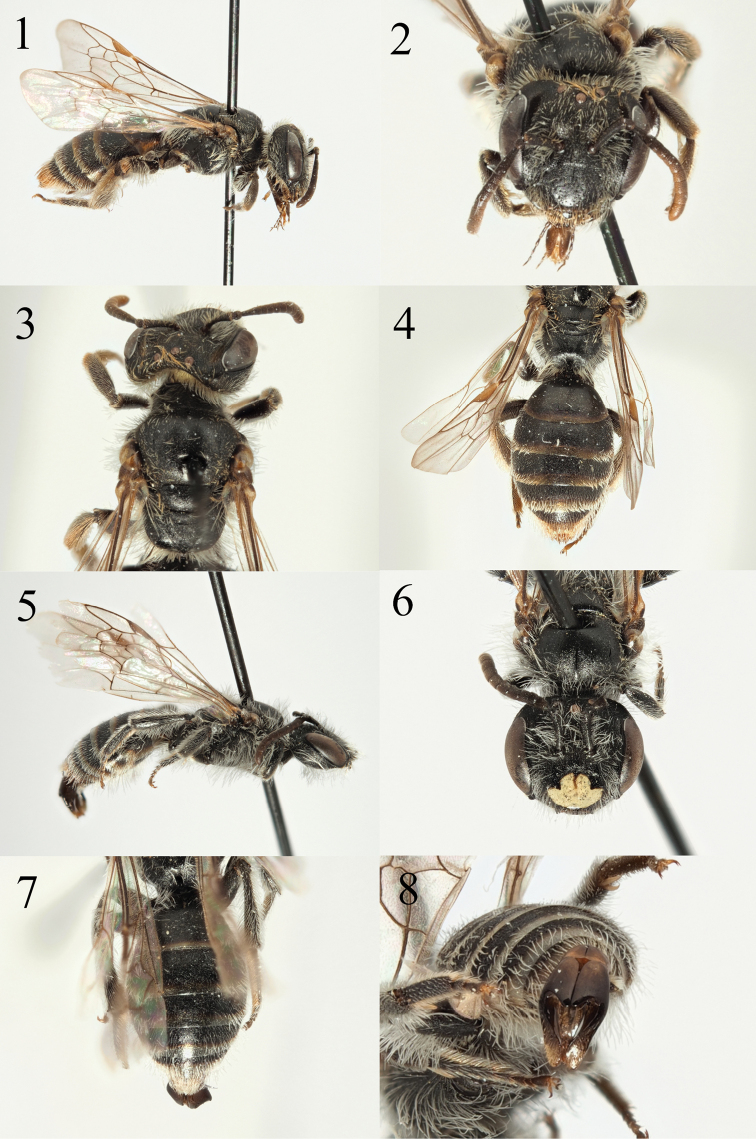
Andrena (Aciandrena) semiadesus sp. nov. **1** female profile **2** female face **3** female dorsum **4** female tergites **5** male profile **6** male face **7** male tergites **8** male genitalia.

**Female**: Body length 7 mm (Fig. 1). ***Head***: Black, as wide as long (Fig. 2). Clypeus broad, slightly arched, dull, strongly shagreened with exception of apical margin where shagreenation is weaker, therefore weakly shining. Sparsely and shallowly punctured, punctures separated by 2–3 puncture diameters. Process of labrum semi-circular, weakly shining. Foveae in lower half deep, narrow, less than half the width of an antenna, very close to inner margin of compound eye. In upper half foveae widen to approximately the width of an antenna, at their widest still occupying less than half the distance between top of compound eye and lateral ocellus. Face, gena, and vertex with moderate brownish to whitish hairs, the longest of these roughly equal to ½ the length of the scape. Antennae dark, A5–12 lightened orange below, A3 equalling A4+5 combined. Gena slightly wider than width of compound eye, weakly longitudinally striate. Ocelloccipital distance short, less than 1/3 width of lateral ocellus. ***Mesosoma***: Scutum and scutellum completely shagreened, weakly shining, punctures shallow and inconspicuous, punctures separated by 2–3 puncture diameters (Fig. 3). Propodeal triangle differentiated from rest of propodeum by larger and coarser shagreenation, weakly shining. Episternum and propodeum finely shagreened, dull. Episternum with sparse, long white hairs, these approaching ¾ of the length of the scape. Legs dark, tarsal segments becoming dark brown. Femoral and tibial scopa simple, white. Wings hyaline, venation light brown, stigma pale yellow, nervulus interstitial to slightly antefurcal. ***Metasoma***: Tergites dark, margins lightened yellow (Fig. 4). Tergal discs strongly microreticulate, punctation extremely sparse and obscured. T2–4 laterally with very weak fringes of white hairs. T5–6 with golden hairs flanking pygidial plate, this rounded with raised margin, shagreened, weakly shining.

**Male.** Body length 6.5–7 mm (Fig. 5). ***Head***: Black, wider than long. Clypeus slightly arched, ground colour black but with large yellow marking covering approximately 90% of surface (Fig. 6). Yellow marking does not extend to base of clypeus. Laterad of clypeal centre, yellow marking reduced, two black triangular markings extending in towards the centre, giving marking a broad, inverted T-shape. In basal half, clypeus dull and shagreened, becoming shiny in apical half. Clypeal punctures shallow and irregular, separated by 2–5 puncture diameters. Process of labrum rectangular, twice as broad and long, shiny. Face, gena, and vertex with long white hairs, longest on underside of head, equalling length of the scape. Scape and pedicel black, following antennal segments dark brown to black, A3 longer than A4, shorter than A4+5 combined. Gena and ocelloccipital distance as in female. ***Mesosoma***: Scutum and scutellum dull, strongly shagreened, sparsely and shallowly punctured, punctures separated by 1–4 puncture diameters. Propodeal triangle weakly marked, scarcely differentiated from propodeum, shagreened, slightly shining laterally. Mesosoma with white hairs, sparse on scutum, denser and longer on episternum, exceeding length of scape. Legs dark, only final tarsal segment slightly lightened to dark brown, pubescence white. Wings hyaline, venation brown, stigma brown, nervulus interstitial. ***Metasoma***: Tergites as in female, but margins more strongly lightened, transparent whitish apically (Fig. 7). Tergal margins laterally with loose white hair bands of irregular length, very widely interrupted. Genitalia simple (Fig. 8), gonocoxites forming rounded dorsal lobes, gonostyli long, tapering to rounded end.

##### Distribution.

Central and eastern Morocco (Fig. 145a).

##### Floral preferences.

None recorded.

##### Etymology.

The name *semi* (partly or partially) + *adesus* (eaten, worn, eroded) was chosen to illustrate the clypeus of the male, where the central yellow marking is laterally diminished by intruding black marks.

#### Andrena (Aciandrena) triangulivalvis

Taxon classificationAnimaliaHymenopteraAndrenidae

Wood sp. nov.

6A67092B-35E3-5750-9998-0953184FEDCA

http://zoobank.org/FE3F09DD-2181-4E54-BA70-FEAABAAA6F52

Figures 9–12

##### Material.

***Holotype***: Morocco: Drâa-Tafilalet, 20 km W Boudnib, 9.iv.1995, 1♂, leg. Ma. Halada. Deposited in the OÖLM. ***Paratypes***: Morocco: Drâa-Tafilalet, 20 km W Boudnib, 9.iv.1995, 3♂, leg. Ma. Halada. Paratypes are deposited at the OÖLM, with a male retained in the personal collection of TJW.

##### Diagnosis.

The male of *A. triangulivalvis* resembles other *Aciandrena* with a yellow polished clypeus such as *A. pratincola*. However, it can be instantly separated from all other *Aciandrena* with a yellow clypeus by the structure of the genitalia. Normal *Aciandrena* genitalia are simple, with a relatively narrow, unmodified penis valve (Figs 8, 100, 102, 104, 126, 128, 130; Warncke 1968b, 1972, 1974, 1975a; Pisanty et al. 2016). However, in *A. triangulivalvis* the penis valve is inflated and forms a triangle that sites between the broad gonostyli that have a slightly raised internal margin (Fig. 12).

##### Description.

**Female**: Unknown.

**Figures 9–12.**
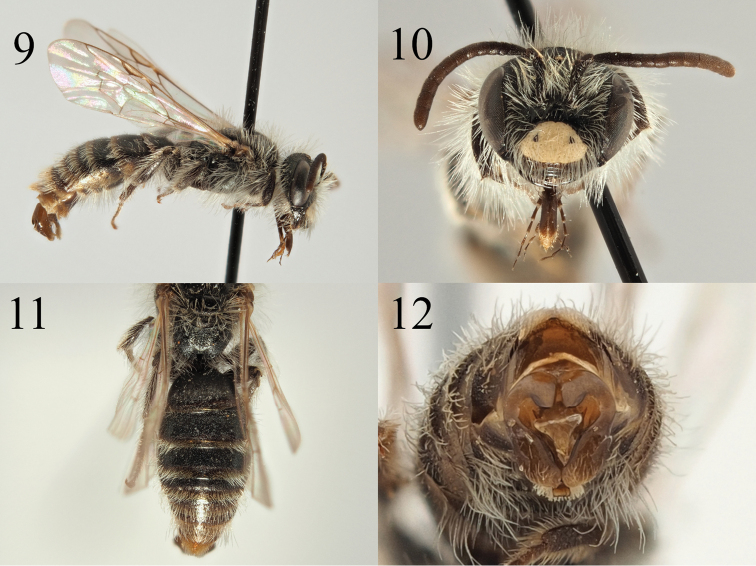
Andrena (Aciandrena) triangulivalvis sp. nov. **9** male profile **10** male face **11** male tergites **12** male genitalia.

**Male.** Body length 6 mm (Fig. 9). ***Head***: Dark, close to black, instead a subtle faintly metallic dark green. Clypeus arched, completely pale yellow with the exception of two lateral small black triangular marks, polished and evenly punctured, punctures separated by 1–2 puncture diameters (Fig. 10). Process of labrum trapezoidal, fore margin emarginate. Gena as wide as width of compound eye. Face, gena, scape, and vertex with long white hairs, the longest achieving length of the scape. Antennae dark, A6–13 slightly lightened brown, A3 equalling A4+5. Ocelloccipital distance short, less than 1/3 width of lateral ocellus. ***Mesosoma***: Scutum and scutellum dark, with slight metallic green-purple hints when viewed from an angle, strongly shagreened, weakly shining, sparsely and irregularly punctured, punctures separated by 1–3 puncture diameters. Propodeal triangle weakly marked, shagreened, slightly shining laterally, centrally weakly rugose. Episternum and propodeum strongly shagreened, obscurely punctured, dull. Scutum, scutellum, episternum, and propodeum with long white hairs, the longest exceeding the scape in length. Legs dark, tarsi lightened to brown, pubescence whitish. Wings hyaline, venation and stigma light brown, nervulus slightly antefurcal. ***Metasoma***: Tergites dark, marginal areas light brown to yellow (Fig. 11). Tergal discs microreticulate, intensity of microreticulation diminishes from T1 onwards, T1 therefore dull, T4–5 weakly shining. T2–4 with weak lateral hair fringes, those on T5–6 extend across the whole margin but sparsely, never obscuring underlying surface. Genitalia compact, slightly longer than wide, gonocoxites forming pronounced points, penis valve strongly inflated basolaterally, forming a triangle (Fig. 12). Gonostyli with inner margin thickened and raised, medially forming a 135-degree angle, gonostyli apexes slightly restricted before forming rounded point.

##### Distribution.

Eastern Morocco from the province of Drâa-Tafilalet (Fig. 145b).

##### Floral preferences.

None recorded.

##### Etymology.

The name *trianguli* (triangular) + *valvis* (valve) was chosen because of the remarkable male genitalia in which the penis valve is inflated and triangular in shape, strongly contrasting with other *Aciandrena* species.

#### Andrena (Campylogaster) sparsipunctata

Taxon classificationAnimaliaHymenopteraAndrenidae

Wood sp. nov.

94749B5E-AF80-59BF-B815-E88CFF11E515

http://zoobank.org/710F0923-3C28-4B47-B6C5-95F4CC4CD880

Figures 13–20 , 21–28

##### Material.

***Holotype***: Morocco: Guelmim-Oued Noun, 10 km E Guelmim, 15–16.iv.1995, 1♀, leg. Ma. Halada. Deposited in the OÖLM. ***Paratypes***: Morocco: Guelmim-Oued Noun, 10 km E Guelmim, 15–16.iv.1995, 1♂, 1♀, leg. Ma. Halada, OÖLM; Oriental, 10 km S Bouarfa, 20.v.1995, 2♂, leg. Ma. Halada, OÖLM. Paratypes are deposited in the OÖLM.

##### Diagnosis.

*Andrena sparsipunctata* can be easily placed into the *Campylogaster* because of the large, dense, and clear punctures on the episternum combined with its large body size. However, recent evidence shows that *Campylogaster* is strongly polyphyletic, and the species in northwestern Africa do not fall close to A. (Campylogaster) erberi Morawitz, 1871, the type species of *Campylogaster* that differs by its tormentose pilosity. A new subgenus is probably needed for the species around *A. sparsipunctata* (Pisanty et al. 2020). Against this context, it is close to the two most widespread *Campylogaster* species in North Africa, A. pruinosa Erichson, 1835 ssp. succinea Dours, 1872 and *A. caroli* Pérez, 1895, both of which are also stained red over the majority of the metasoma. The female of *A. sparsipunctata* is instantly recognisable because the punctures of the first tergite are sparse, separated by 2–4 puncture diameters (Fig. 16) whereas in the other two species the punctures are dense and separated by 1–2 puncture diameters (Figs 18, 20). Moreover, the foveae of *A. sparsipunctata* females are narrow and depart from the inner eye margins so that at the top of the compound eye they are separated from the compound eye by a distance subequal to the width of a fovea itself (Fig. 15), whereas in the other two species the foveae are never clearly separated from the top of the compound eye (Figs 17, 19). The scutal hairs are also normal, not squamous (Fig. 15), strongly contrasting with the squamous hairs of the other two species (Figs 17, 19).

**Figures 13–20.**
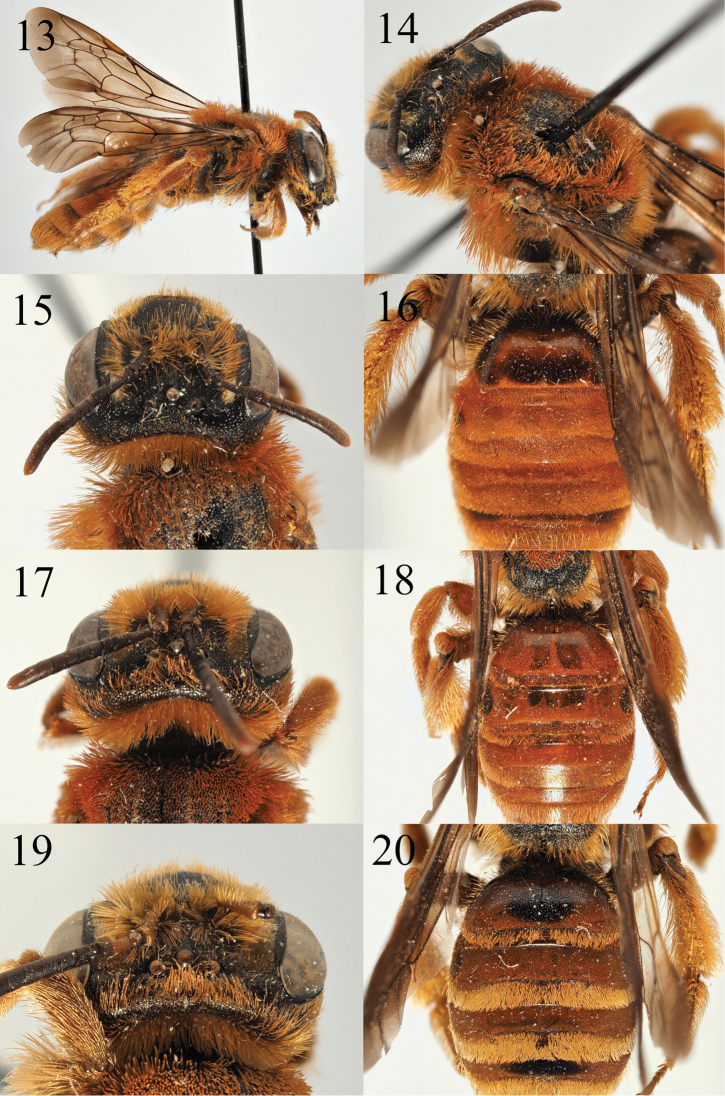
Andrena (Campylogaster) sparsipunctata sp. nov. **13** female profile **14** female mesonotum in semi-profile **15** female head dorsal view **16** female tergites. Andrena (Campylogaster) pruinosa succinea Dours, 1872 **17** female head dorsal view **18** female tergites. *Andrena caroli* Pérez, 1895 **19** female head dorsal view **20** female tergites.

In males, *A. sparsipunctata* can be recognised by the shape of the labrum which is wide and rectangular (Fig. 23), whereas in *A. pruinosa* it is trapezoidal and markedly emarginate (Fig. 25). In *A. caroli* it is less strongly trapezoidal and emarginate (Fig. 27), but the tergites have well-marked and dense bands of short white hairs on the tergal margins (Fig. 28), whereas *A. sparsipunctata* has only a few scattered hairs on the tergal margins, never forming well-marked bands, as in *A. pruinosa* (Fig. 26).

**Figures 21–28.**
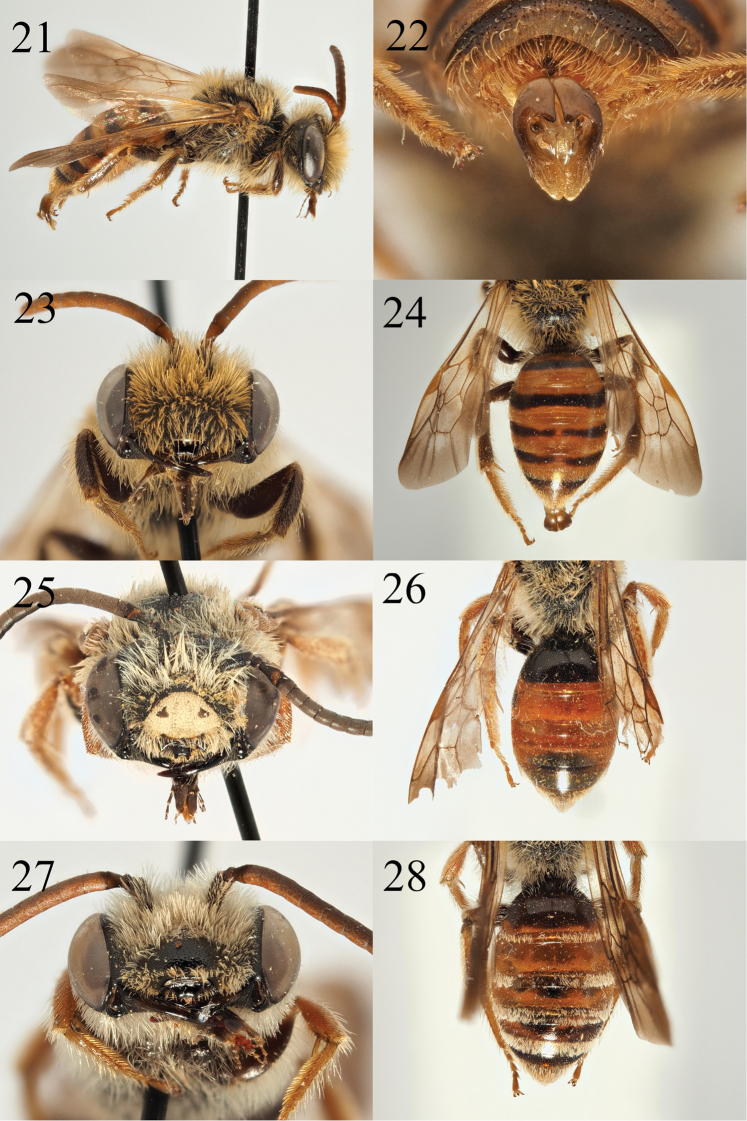
Andrena (Campylogaster) sparsipunctata sp. nov. **21** male profile **22** male genitalia **23** male face and labrum **24** male tergites. Andrena (Campylogaster) pruinosa succinea Dours, 1872 **25** male face and labrum **26** male tergites. *Andrena caroli* Pérez, 1895 **27** male face and labrum **28** male tergites.

##### Description.

**Female**: Body length 12 mm (Fig. 13). ***Head***: Black, a little wider than long. Clypeus domed, evenly and shallowly punctured, punctures separated by 0.5–1 puncture diameters. Clypeus surface weakly shining, fore margin slightly upturned. Process of labrum trapezoidal, with weak lateral striations, fore margin weakly and narrowly emarginate. Gena 1.2 times wider than width of compound eye, densely and deeply punctate, punctures extend to the vertex where they become much shallower and encircle two impunctate areas immediately adjacent to lateral ocelli, these areas equivalent to inter-ocellar area in size (Fig. 15). Gena and vertex with a mane of bright ginger hairs, achieving a maximum length of 2/3^rds^ of the length of the scape. Face, scape, and clypeus with shorter ginger hairs, these achieving a maximum length of ½ the length of the scape. Antennae dark, A3 apically and A4–12 lightened orange below, A3 longer than A4+5, shorter than A4+5+6. Foveae narrow, slightly narrower than the width of an antenna, close to the internal margin of compound eye below level of antennal insertions but diverging above so that at top of compound eye each fovea is separated by a distance subequal to its own width, area between a fovea and the internal margin of compound eye shiny and sparsely punctured. Ocelloccipital distance broad, two times the width of lateral ocellus. ***Mesosoma***: Scutal punctures dense, punctures almost touching, underlying surface shiny. Scutum and scutellum with non-squamous hairs, these achieving a maximum length of ½ the length of the scape (Fig. 14). Episternum and propodeum with exception of propodeal triangle densely and evenly punctate, punctures separated by 0.5 puncture diameters, underlying surface finely and subtly shagreened, weakly shining. Propodeal triangle weakly rugose. Episternum and propodeum with normal ginger hairs, these achieving the length of the scape. Femora dark, tibia and tarsi orange. Femoral and tibial scopa orange. Wings slightly infuscate, venation dark, stigma slightly translucent. Nervulus interstitial. ***Metasoma***: Tergites amber, becoming amber to dark brown on T1. T1 strongly contrasting with following tergites, T1 sparsely punctate, punctures separated by 2–4 puncture diameters, surface of disc clear of hairs, shining (Fig. 16). T2–4 densely punctured, punctures separated by 0.5–1 puncture diameters, covered in short ginger hair across entire surface. Tergal margins slightly depressed, covered in short ginger hairs that form weak apical fringes, poorly differentiated from hairs on discs of T2–4. T5–6 with long ginger-golden hairs flanking pygidial plate, pygidial plate simple, triangular, without raised margins.

**Male.** Body length 11–12 mm (Fig. 21). ***Head***: Similar to female, wider than long, pubescence whitish throughout but probably faded. All punctation weaker and finer with exception of clypeus where it is equally strong as in the female. Labrum broadly rectangular, wider than long, fore margin straight, without emargination (Fig. 23). Mandibles long, crossing, very slightly bidentate with tiny internal tooth. Scape dark-orange, A2–13 evenly orange coloured, A3 equalling A4. Ocelloccipital distance broad, 1.5 times width of lateral ocellus. ***Mesosoma***: Similar to female, punctation of the scutum and scutellum weaker, punctation of the episternum and propodeum absent, replaced by weak rugosity. Venation of wings amber, nervulus interstitial to antefurcal. ***Metasoma***: Tergites amber, extent variable, becoming darker on centre of tergal discs, in some cases forming extensive dark bands across entire surface (Fig. 24). Punctation shallow and even, separated by 1–2 puncture diameters, not noticeably sparser on T1 compared to other tergites. Sternite 8 long, rectangular, three times longer than broad, covered in hairs, apex weakly emarginate. Genitalia simple (Fig. 22), gonocoxites slightly pointed, weakly separated from each other apically.

##### Distribution.

Southern and eastern Morocco in desert environments (Fig. 145c).

##### Floral preferences.

None recorded.

##### Remarks.

There is uncertainty over the exact status of *A. pruinosa succinea*, as the North African animals differ in colouration of the metasoma and scutal hairs (red) from *A. pruinosasensu stricto* (black-brown) from Spain. However, structural differences are minor (e.g., the shape of the foveae is the same), and molecular investigation is warranted. It is however clear that the name *succinea* cannot apply to the bees described here as the first tergite of females is much less densely punctured than *A. pruinosa* s.l. from either Spain or North Africa, and Dours (1872) makes no mention of contrasting tergal punctation in his description ‘Abdomen… très-finement ponctué et hérissé de poils…’. The sampling locations of *A. sparsipunctata*, both found at the extreme edges of Morocco, may provide an explanation as to why this species has gone undetected to date.

##### Etymology.

The name *sparsi* (sparse) + *punctata* (punctured) was chosen to illustrate the first tergite of females, which is much less densely punctate than the similarly coloured *A. pruinosa succinea* and *A. caroli*.

#### Andrena (Carandrena) hebescens

Taxon classificationAnimaliaHymenopteraAndrenidae

Wood sp. nov.

E9D30E8D-5F43-5192-A6E9-7BAD7E7EA872

http://zoobank.org/BECF6642-E2D6-479D-A132-18F49B9725B6

Figures 29–34 , 35–40

##### Material.

***Holotype***: Morocco: Guelmim-Oued Noun, 15–16.iv.1995, 1♀, leg. Ma. Halada. Deposited in the OÖLM. ***Paratypes***: Morocco: Guelmim-Oued Noun, 15–16.iv.1995, 4♂, 6♀, leg. Ma. Halada, OÖLM; Souss-Massa, Tassademt, 50 km NE, Agadir, 19.iv.1996, 1♀, leg. M. Schwarz, OÖLM; Souss-Massa, Aoulouz-Taliouine, 19.iii.1988, 1♂, leg. H. Teunissen. Paratypes are deposited at the OÖLM and NMNL, with a male and female retained in the personal collection of TJW.

##### Diagnosis.

The female of *A. hebescens* can be placed in the *Carandrena* because the dorsolateral angle of the pronotum has a transverse ridge, the propodeal triangle is shagreened and weakly rugose at the base, and there is almost no punctation on the metasoma, and the head has a typical *Carandrena* shape, broader than long, with the inner eye margins slightly converging below (Fig. 30). It is most similar to *A. euzona* Pérez, 1895 and *A. microthorax* Pérez, 1895 given its dark, non-metallic appearance (*A. aerinifrons* Dours, 1873, *A. bellidis* Pérez, 1895, *A. daphanea* Warncke, 1974, *A. deserta* Warncke, 1974, *A. nigroviridiula* Dours, 1873, and *A. reperta*, Warncke, 1974 with metallic green or blue integument, *A. binominata* Smith, 1853, *A. eremobia* Guiglia, 1933, and *A. leucophaea* Lepeletier, 1841 with partially red metasoma), dull domed clypeus, and white hair bands on the tergites (*A. eddaensis* Gusenleitner, 1998 and *A. decaocta* Warncke, 1967 with felt-like hair on the tergal discs). However, the scutum is shagreened and only weakly shining (Fig. 31) whereas in *A. euzona* it is extensively smooth and shiny (Fig. 33), and the hair bands are narrower (Fig. 32) whereas in *A. euzona* they are wider (Fig. 34). The clypeus is also evenly punctured and shagreened, weakly shining with only a subtle impunctate mid-line whereas in *A. microthorax* it is shiny and strongly punctured with a conspicuous impunctate mid-line, particularly at the fore margin of the clypeus where it forms a broad impunctate triangle.

**Figures 29–34.**
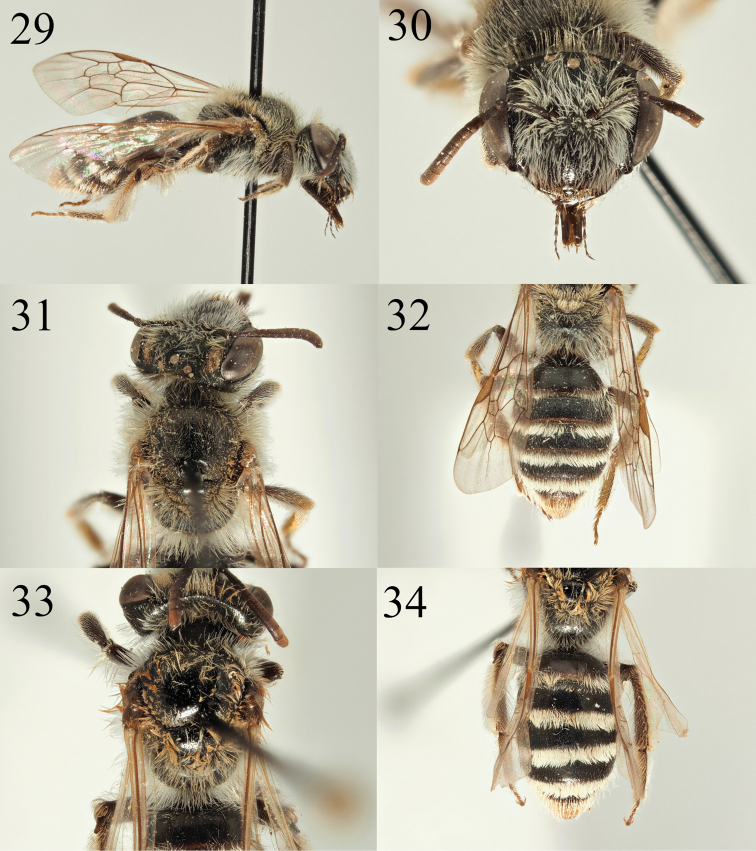
Andrena (Carandrena) hebescens sp. nov. **29** female profile **30** female face **31** female dorsum **32** female tergites. Andrena (Carandrena) euzona Pérez, 1895 **33** female dorsum **34** female tergites.

The male of *A. hebescens* has a yellow marking on the clypeus, but this is reduced in size and does not cover the entire clypeal surface, with two black markings that extend in from the clypeal margins giving the marking an inverted ‘T’ shape (Fig. 36). The male is therefore superficially similar to *A. semiadesus*, but can be separated by the typical *Carandrena* head which is clearly wider (more than 1.5 times) than the width of a compound eye in *A. hebescens* (Figs 37, 38) but only slightly wider in *A. semiadesus* and lacking an angulate hind corner. The male of *A. microthorax* can have a yellow clypeal marking, but the scutum and tergites of this species are shiny, whereas in *A. hebescens* they are shagreened and at most, weakly shining (Fig. 39).

##### Description.

**Female**: Body length 8 mm (Fig. 29). ***Head***: Black, clearly wider than long (Fig. 30). Clypeus arched, shagreened, weakly shining. Clypeus evenly punctured with exception of a subtle, impunctate midline, punctures otherwise separated by 0.5–1 puncture diameters. Process of labrum trapezoidal, fore margin very weakly emarginate. Gena as wide as width of compound eye. Gena, face, and scape with moderately dense white hairs, the longest not achieving length of the scape. Vertex with whitish brown hairs of a similar length. Foveae normal, occupying half the distance between the compound eye and a lateral ocellus. Antennae dark, scape black, A2–4 apically lightened to orange, A5–12 predominantly orange ventrally, A3 exceeding A4+5, shorter than A4+5+6. Ocelloccipital distance short, less than 1/3 width of lateral ocellus. ***Mesosoma***: Scutum dark, evenly and shallowly punctured, punctures separated by 1–2 puncture diameters, underlying surface shagreened, weakly shining (Fig. 31). Scutellum less densely punctured, punctures separated by 3–4 puncture diameters, shagreenation weaker, more strongly shining. Episternum and propodeum microreticulate, dull, propodeal triangle slightly more finely shagreened, weakly shining, distinct. Scutum and scutellum with faded light brownish hairs, episternum and propodeum with longer white hairs, the longest achieving the length of the scape. Legs dark, tarsi lightened brown, pubescence white, femoral and tibial scopa white, hairs simple. Wings hyaline, venation and stigma golden brown. Nervulus interstitial to slightly antefurcal. ***Metasoma***: Tergites dark with wide lightened margins, apically translucent, basally yellowish (Fig. 32). Tergal discs microreticulate, weakly shining. T1 shallowly punctured, punctures separated by two puncture diameters. Following tergites weakly and obscurely punctured, punctures hidden by microreticulation. Tergal margins with dense white hairbands, on T1 widely interrupted, on T2–4 complete. T5+6 centrally with golden hairs flanking pygidial plate, laterally with white hairs. Sternites with plumose white hairs, forming loose fringes apically on hind margins.

**Figures 35–40.**
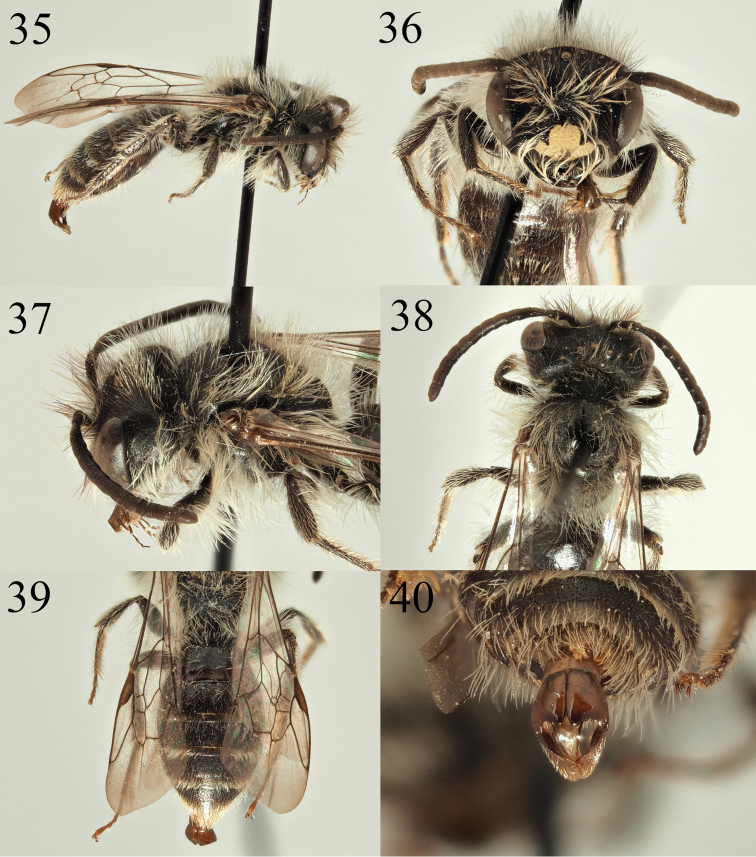
Andrena (Carandrena) hebescens sp. nov. **35** male profile **36** male face **37** male gena **38** male dorsum **39** male tergites **40** male genitalia.

**Male.** Body length 8 mm (Fig. 35). ***Head***: Similar to female, clypeus slightly arched, evenly and shallowly punctured, punctures separated by one puncture diameter, no impunctate central line. Clypeus centrally with yellow mark, this not reaching the lateral or basal margin of the clypeus, laterally invaded by two black marks therefore forming a broad inverted T-shape (Fig. 36). Underlying surface weakly shagreened, moderately shiny. Process of labrum trapezoidal, fore margin inflated, slightly bulbous, very weakly emarginate. Antennae dark, A4–13 slightly lightened dark brown, A3 exceeding A4, shorter than A4+5. Gena enlarged, most 1.2 times wider than compound eye, non-carinate, with weakly angulate hind corner (Fig. 37). Gena, vertex, and face below the level of the antennal insertions with long white hair equalling length of the scape. Scape, frons, and inner margin of compound eyes with mixture of black and white hairs. Ocelloccipital distance short, 2/3 width of lateral ocellus. ***Mesosoma***: Similar to female but scutum with stronger shagreenation, dull except for central shining line (Fig. 38). Scutellum centrally weakly shining, contrasting with the scutum. Episternum, propodeum, and mesosomal pubescence as in the female. Legs dark, tarsi lightened brown, with white pubescence. Wings hyaline, venation dark brown, stigma centrally light brown. Nervulus interstitial to slightly antefurcal. ***Metasoma***: Similar to the female. Tergites more clearly punctured, punctures visible against the microreticulation with slightly raised margins giving the overall surface an uneven impression (Fig. 39). T1–5 consistently punctured, punctures separated by 3–4 puncture diameters. T2–4 laterally with weak fringes of white hair, T5–6 with complete fringes of whitish to golden hairs. Sternites forming loose white hair bands apically. Genitalia simple, of a typical *Carandrena* form, gonocoxites apically forming weak points, rounded, diverging apically (Fig. 40).

##### Distribution.

South-western Morocco in the Souss valley (Fig. 145a).

##### Floral preferences.

None recorded.

##### Etymology.

The name *hebescens* was chosen because this member of the *Carandrena*, though morphologically similar to several species with metallic green colouration, is completely dark, therefore *heb*- (dull or blunt) + *escens* (becoming).

#### Andrena (Cnemidandrena) niveofacies

Taxon classificationAnimaliaHymenopteraAndrenidae

Wood sp. nov.

D6D99A74-2D35-5BAF-B033-52020326E1BD

http://zoobank.org/E8892496-603A-4AD8-BB34-E5F63A35E60E

**Figures 41–46.**
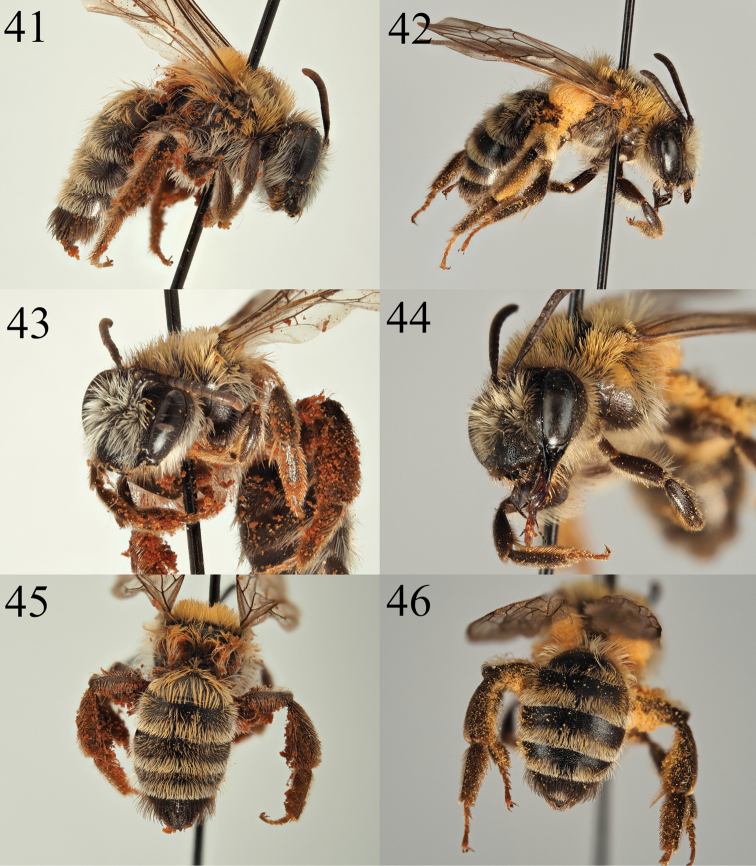
Andrena (Cnemidandrena) niveofacies sp. nov. **41** female profile **43** female face **45** female tergites. Andrena (Cnemidandrena) simillima sischkai Warncke, 1988 (holotype) **42** female profile **44** female face **46** female tergites.

Figure 41–46 , 47–54

##### Material.

***Holotype***: Morocco: Marrakesh-Safi, Asni, S Imlil, 2900 m, 24.viii.1992, 1♀, leg. Warncke [see Remarks]. Deposited in the OÖLM. ***Paratypes***: Morocco: Marrakesh-Safi, Asni, S Imlil, 3300 m, 21.viii.1992, 1♂, leg. Warncke, OÖLM.

##### Diagnosis.

*Andrena niveofacies* can easily be recognised as part of the *Cnemidandrena* because of the transverse ridge on the dorsolateral angle of the pronotum, the triangular hind tibiae in the female, the upturned process of the labrum in the male (Fig. 48), the thick and distinct hair bands on the metasoma (Figs 45, 46), and the late summer activity period.

Within the *Cnemidandrena*, *A. niveofacies* females have completely brown hair on the scutum (intermixed with black in *A. denticulata* (Kirby, 1802)), the galea is dull (shiny in *A. fuscipes* (Kirby, 1802)), T5–6 are black haired (light haired in *A. tridentata* (Kirby, 1802)), and the face is pale haired (black at least in part in *A. nigriceps* (Kirby, 1802) and *A. freygessneri* Alfken, 1904). The female *A. niveofacies* is most similar to *A. simillima* Smith, 1851 but is can be easily separated as it has strikingly bright white hairs on the face and gena (Fig. 43), these being buff-brown in *A. simillima*.

The male has a non-carinate gena (Fig. 49, carinate in *A. denticulata* and *A. tridentata*), the galea is dull (shining in *A. fuscipes*), S8 is short and relatively densely haired (long and sparsely haired in *A. freygessneri*, see illustrations in Ember 2001), and the face has bright white hairs, with no trace of black hairs on the discs of the tergites (face with brown hairs in *A. nigriceps*, T4–5 with black hairs basally). However, the male material also differs from *A. simillima* as A3+4 are equal in length, whereas in *A. simillima* A3 is a little longer than A4.

##### Description.

**Female**: Body length 10 mm (Fig. 41). ***Head***: Black, a little wider than long. Clypeus slightly arched, evenly and clearly punctured, punctures dense, separated by 0.5–1 puncture diameters with the exception of subtle central impunctate line than widens into a triangle immediately before fore margin of clypeus. Underlying surface weakly shagreened, slightly shining. Process of labrum trapezoidal, fore margin clearly emarginate. Gena slightly wider than width of compound eye. Gena, face, and scape with bright white hairs, longest achieving length of the scape (Fig. 43). Vertex with contrasting brown and black hairs of equal length. Foveae generally wide, occupying three quarters of distance between top of compound eye and lateral ocellus. Antennae black, ventral surface of A4–12 lightened brown, A3 slightly shorter than A4+5. Ocelloccipital distance equalling width of lateral ocellus. ***Mesosoma***: Scutum densely punctate, punctures separated by 0.5 puncture diameters over majority of disc but becoming sparser towards rear in centre and on scutellum, here separated by one puncture diameter. Underlying surface microreticulate, but this weakens to become fine shagreenation, shining, centrally and laterally on scutum and particularly on scutellum. Episternum with microreticulation, dull. Propodeum laterally shagreened, weakly shining, posterolaterally microreticulate and dull with large shallow punctures, these absent from the propodeal triangle which is therefore well defined. Scutum, scutellum, and propodeum with buff-brown hairs of moderate length, contrasting with white hairs on episternum. Legs dark, tarsal segments lightened brown. Wings hyaline, venation black, stigma centrally dark brown. Nervulus clearly antefurcal. ***Metasoma***: Tergites black, microreticulate, weakly shining, moderately punctured, punctures separated by 1–2 puncture diameters (Fig. 45). T1 with long buff-brown hairs over whole disc and margin, T2–4 with clear buff-brown hair bands occupying marginal area, surface of disc with sparser and shorter hairs of same colour. T5–6 with black-brown hairs. S2–4 with sparse white hairs forming weak bands.

**Figures 47–54.**
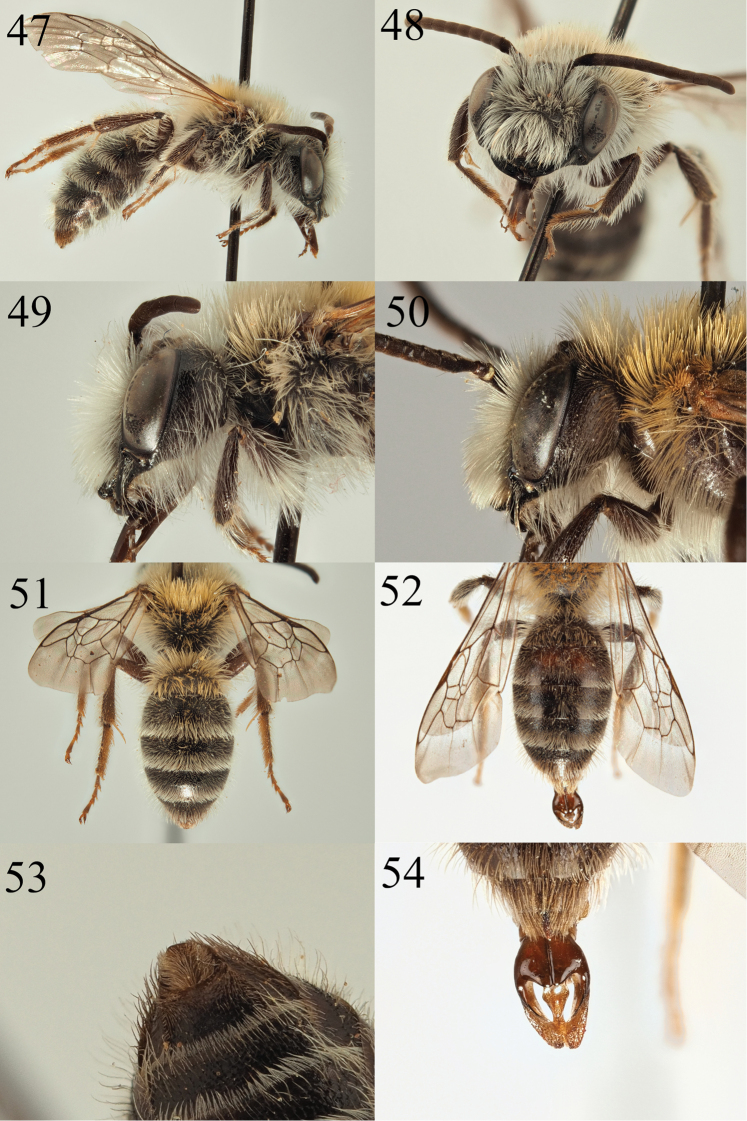
Andrena (Cnemidandrena) niveofacies sp. nov. **47** male profile **48** male face **49** male gena **51** male tergites **53** male sternite 8. Andrena (Cnemidandrena) simillima sischkai Warncke, 1988 (paratype) **50** male gena **52** male tergites **54** male genitalia.

**Male.** Body length 10 mm (Fig. 47). ***Head***: As in the female, but clypeus without central impunctate line, process of labrum narrow and centrally upturned (Fig. 48). Galea dull (Fig. 49). Antennae dark, A4–13 lightened to dark brown below, A3 equalling A4. Gena wide, 1.5 times wider than width of compound eye, non-carinate, forming weak posterior angle. Face, gena, scape, and vertex all with long white hairs achieving length of the scape. Ocelloccipital distance broad, 1.5 times width of lateral ocellus. ***Mesosoma***: Scutum and scutellum as in female, microreticulation weaker so the whole surface appears shinier. Propodeal punctures weaker and shallower, slightly obscured by weak reticulation, contrast with the propodeal triangle as in the female. ***Metasoma***: As in female (Fig. 51), T2–5 with pale hair bands, T6 with brown apical fringe. S8 short and densely hairy (Fig. 53).

##### Distribution.

Probably restricted to the High Atlas Mountains of Morocco. This material represents the first record of the subgenus Cnemidandrena from Morocco and more broadly the whole of North Africa (Fig. 145b).

##### Floral preferences.

The single female had a full scopa (Fig. 43) comprising 95% *Cirsium*-type (Asteraceae: Cynareae) and 5% *Eryngium*-type (Apiaceae). It is too early to draw strong conclusions, as *Cnemidandrena* contain both oligolectic (*A. denticulata*, Asteraceae, Wood and Roberts 2017) and polylectic species (*A. freygessneri*, *A. nigriceps*, *A. simillima*; Wood and Roberts 2017; Else and Edwards 2018; Müller 2018).

##### Remarks.

There has historically been uncertainty over the species status of taxa assigned to *A. simillima*. The former subspecies *A. freygessneri* was returned to species status by Ebmer (2001), but the status of *A. s. bremensis* Alfken, 1900 and *A. s. sischkai* Warncke, 1988 remain unclear. This latter subspecies is found in Bulgaria and Greece (Figs 42, 44, 46, 50, 52, 54; Warncke 1988) and the facial hairs are buff coloured as in the nominate form (Fig. 44), but the tergites are much less densely hairy and the hairs are shorter, giving the overall impression that the bee is darker (Figs 46, 52). It is likely that both taxa are distinct from *A. simillima*, but more work is required. Against this context, we describe *A. niveofacies* as a good species, not only because of the morphological differences but also because of the degree of geographical separation, with the *locus typicus* in the High Atlas approximately 1,600 km from the nearest *A. simillima* records in southern France.

The collection labels themselves are a small mystery, as they have the collector name Warncke struck through by hand (Warncke). They may have been used by a collector accompanying Warncke who used his spare preprinted labels, but if this is the case then their identity is not clear.

##### Etymology.

The subspecific epithet *niveofacies* from *niveus* (snow) + *facies* (face) was chosen to illustrate the bright white facial hair.

##### Other material examined.

(*Andrena simillima sischkai*): Greece: Olympus, 2000–2200 m, 18.viii.1978, 1♀, leg. K. Warncke, OÖLM (holotype); Bulgaria: mer., Pirin, Popina-Lka I (1350 m), 23–27.vii.1974, 1♂, leg. Dr. A. Hoffer, OÖLM (paratype); (*Andrena simillima simillima*): United Kingdom: Devon, Weston Cliff NT, Branscombe, 23.vii.1992, 1♀, leg. M. Edwards, TJW.

#### Andrena

Taxon classificationAnimaliaHymenopteraAndrenidae

(incertae sedis) gafsensis Wood sp. nov.

F19DDE05-D0B6-505A-9AE9-0202C6C8DA9B

http://zoobank.org/B4DDA0DB-D0BC-4675-B135-B20636594D00

Figures 55–62

##### Material.

***Holotype***: Tunisia: 40 km NW Gafsa, 17.iv.1994, 1♀, leg. M. Schwarz. Deposited in the OÖLM. ***Paratypes***: Tunisia: 40 km NW Gafsa, 17.iv.1994, 3♂, 1♀, leg. M. Schwarz. Paratypes are deposited at the OÖLM, with a male and female retained in the personal collection of TJW.

##### Diagnosis.

Placement of this species into a subgenus is not immediately obvious. *Andrena gafsensis* is a small to medium sized *Andrena* with a strongly flattened clypeus that is extensively shiny in both the female (Fig. 56) and the male where it is coloured yellow, with this colouration extending onto the lower paraocular areas (Fig. 60). This pattern of yellow colouration is typically found in males from the subgenera *Holandrena* Pérez, 1980, *Ulandrena* Warncke, 1968, and *Nobandrena* Warncke, 1968. Specimens can quickly be separated from the first two because of the sculpturing of the episternum and propodeum (simple, not honeycomb-areolate) and from the second by the shape of the female hind tibial spur (straight not medially broadened) and the genitalia of the male (penis valve not inflated).

*Nobandrena* normally contains large species in the range of 13–14 mm in length, most of which have males with domed clypei which are extensively yellow-marked, with the yellow extending onto the lower paraocular areas. However, there are two smaller species *A. iliaca* Warncke, 1969 (7–8 mm, Turkey, Israel, and Jordan) and *A. ounifa* Warncke, 1974 (7–7.5 mm, Algeria, see below for new description of female) placed into the *Nobandrena* by Warncke where the male clypeus is shinier and flatter, whilst retaining the same colour pattern. This flattened clypeus can be seen in the female of *A. ounifa* which has a strongly flattened and shining clypeus in both the female (Fig. 94) and the male (Fig. 98). However, these two taxa clearly do not belong in the *Nobandrena*, with molecular analysis placing *A. iliaca* in the *Fuscandrena* (Pisanty et al. 2020). The position of *A. ounifa* is unclear without molecular work, and so confident placement of *A. gafsensis* is not possible at this time.

Given these problems, *Andrena gafsensis* is therefore best recognised in the female by the distinctive clypeal structure (Fig. 56), but then separated from *A. ounifa* by the short propodeum (Fig. 57) and by the colouration of the tergites which are almost completely red except for the basal parts of T1 and two lateral black spots on T2 (Fig. 58). The male can be recognised by the clypeal structure and colouration, but can also be separated by the short propodeum as in the female and by the structure of the genitalia (Fig. 62) which are most similar to *A. iliaca* but the gonocoxites form lateral points and the gonostyli lack an emargination in the outer margin.

**Figures 55–62.**
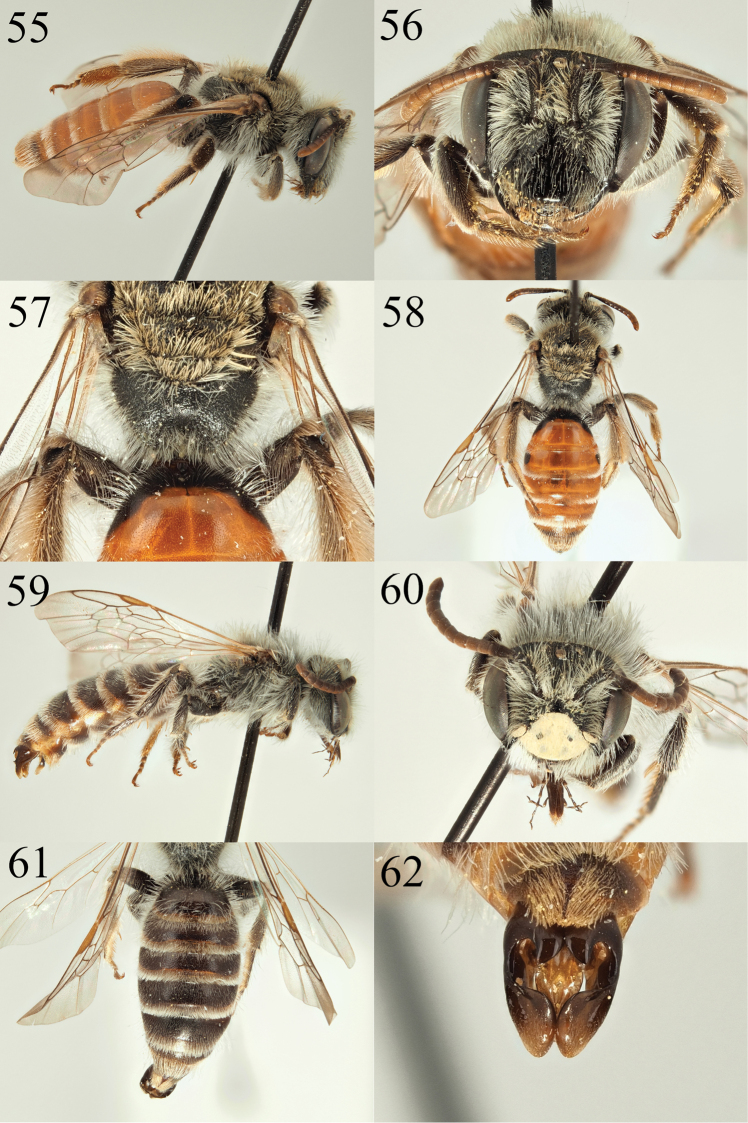
Andrena (Nobandrena) gafsensis sp. nov. **55** female profile **56** female face **57** female propodeum **58** female tergites **59** male profile **60** male face **61** male tergites **62** male genitalia.

##### Description.

**Female**: Body length 9 mm (Fig. 55). ***Head***: Black, as wide as long. Clypeus very slightly arched laterally, flattened over the majority of its area (Fig. 56). Laterally with punctures separated by one puncture diameter but only immediately adjacent to clypeal margin, remaining parts more sparsely punctured, punctures separated by 2–3 puncture diameters, underlying surface smooth and shiny. Process of labrum broad, trapezoidal, approximately twice as wide basally as long. Gena approximately width of compound eye. Gena, vertex, face, and scape with white hairs, the longest not exceeding length of the scape. Foveae of average width, occupying just over half the distance between top of compound eye and lateral ocellus. Antennae black, A4–12 extensively lightened orange below, A3 equalling A4+5+6. Ocelloccipital distance short, less than 1/3 width of lateral ocellus. ***Mesosoma***: Scutum and scutellum densely punctured, punctures separated by 1–1.5 puncture diameters, underlying surface of the scutum shagreened anteriorly and laterally, shiny centrally, posteriorly and on scutellum. Episternum microreticulate, dull. Lateral faces of propodeum (propodeal corbicula) shagreened, weakly shining. Hind face of propodeum microreticulate and weakly rugose, rugosity outlining propodeal triangle which is rugose and therefore defined by change in texture (Fig. 57). Scutum and scutellum with short brown hair, at most achieving the length of A3. Episternum and propodeum with longer white hair, never exceeding length of the scape. Legs dark, tarsi lightened brown, pubescence brown to brownish white. Femoral scopa white, tibial scopa white to light brown. Wings hyaline, venation brown, stigma light brown. Nervulus slightly antefurcal. ***Metasoma***: Tergites light red with exception of black basal and lateral parts of T1 and two black lateral spots on T2, and apex of T5 which is slightly lightened brown (Fig. 58). Sternites light red with exception of S1 and apical margins of S4–6. Tergites densely, finely, and evenly punctured, punctures separated by one puncture diameter. T2–4 with thin white hair bands of fine hairs, on T2–3 widely interrupted, on T4 complete. T5–6 with golden hairs flanking pygidial plate, laterally white. Pygidial plate with slightly raised margins, centrally with longitudinal slightly raised area.

**Male.** Body length 9–10 mm (Fig. 59). ***Head***: Black, slightly wider than long. Clypeus very shallowly arched, essentially flat, ivory-coloured with exception of two faint dark spots centrally, yellow colouration extending onto bottom parts of paraocular areas (Fig. 60). Underlying clypeal surface shiny, moderately and shallowly punctured, punctures separated by 1–2 puncture diameters. Process of labrum trapezoidal, fore margin broadly and shallowly emarginate, shiny. Pubescence and antennal colouration as in female, A3 equalling A4+5. Ocelloccipital distance slightly shorter than width of lateral ocellus. ***Mesosoma***: Scutum and scutellum dark, shagreened, dull laterally and anteriorly, weakly shining only centrally, moderately punctured, punctures separated by one puncture diameter. Sculpturing of episternum and propodeum as in female. Pubescence as in female. Wings hyaline, venation brown, stigma light brown. Nervulus clearly antefurcal. ***Metasoma***: Tergites predominantly dark, tergal margins slightly depressed, lightened yellow to orange (Fig. 61). Tergites microreticulate, weakly shining, shallowly punctured, punctures separated by 1–2 puncture diameters. T2–5 with loose white hair fringes, that on T2 weakly interrupted, the following complete. T6 with fringe of longer white-golden hairs. Genitalia simple, gonocoxites diverging apically and forming points (Fig. 62). Sternite 8 short, densely hairy.

##### Distribution.

Known only from the *locus typicus* in southern Tunisia.

##### Floral preferences.

None recorded.

##### Etymology.

Named after the nearest indicated town Gafsa in southern Tunisia.

#### Andrena

Taxon classificationAnimaliaHymenopteraAndrenidae

(incertae sedis) tenebricorpus Wood sp. nov.

05CF51C2-0CB9-5B71-AF9A-0DFFE6251846

http://zoobank.org/6C1EA635-A7DD-418E-BC59-CF97AB02F508

Figures 63–66

##### Material.

***Holotype***: Morocco: Guelmim-Oued Noun, 10 km E Guelmim, 15.iv.1995, 1♀, leg. Ma. Halada. Deposited in the OÖLM.

##### Diagnosis.

**Figures 63–66.**
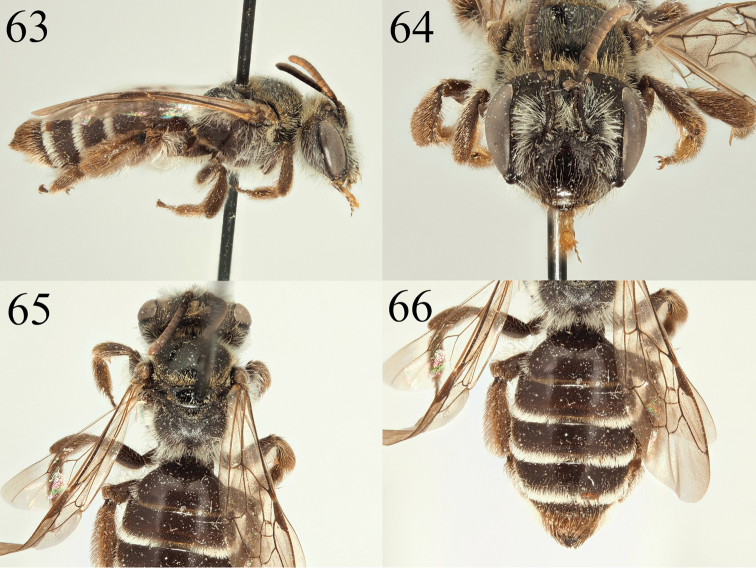
Andrena (Nobandrena) tenebricorpus sp. nov. **63** female profile **64** female face **65** female propodeum **66** female tergites.

*Andrena tenebricorpus* is very similar to *A. gafsensis* and faces the same problems of subgeneric classification, and is therefore also not currently placed in one until molecular data are available. Both species have a flattened and shiny clypeus (Fig. 64) and a short propodeum (Fig. 65), but differ most obviously in colour, with *A. tenebricorpus* having dark brown terga (Fig. 66) in strong contrast to the red terga of *A. gafsensis*. In terms of structural difference, in *A. tenebricorpus* the foveae are slightly wider, the hairs on the scutum and scutellum are relatively much shorter (compare Figs 57, 65) at the same time that the hair bands on the apical margins of the tergites are relatively much thicker (compare Figs 58, 66), suggesting that this is not as a result of abrasion, and the scopa are a dirty brown (Fig. 63) rather than white (Fig. 55).

In terms of overall colouration and appearance *A. tenebricorpus* is extremely similar to *A. guichardi* Warncke, 1980 (also found only in south-western Morocco) with the same dark to dark brownish tergal colouration with contrasting white hair bands (see Fig. 104). However, *A. tenebricorpus* can be clearly separated by the flattened clypeus (domed in *A. guichardi*), more densely punctate scutum (punctures separated by 1–2 puncture diameters, by 2–4 puncture diameters in *A. guichardi*, see Fig. 103), and by the propodeal triangle which is weakly differentiated only by sculpturing (clearly marked by a small raised carina in *A. guichardi*).

##### Description.

**Female**: Body length 10 mm (Fig. 63). ***Head***: Dark, as wide as long. Clypeus slightly arched laterally, broadly flattened on the disc, underlying surface laterally with shagreenation, the majority of disc shiny (Fig. 64). Moderately punctured, punctures separated by 1–2 puncture diameters, with generally impunctate line in centre of clypeus. Process of labrum trapezoidal with faint lateral striations, fore margin weakly emarginate, slightly bulging. Gena equal to width of compound eye. Gena, face, and scape with white hairs, longest not exceeding length of the scape. Vertex with mixture of whitish and yellowish hairs of same length as on rest of face. Foveae of average width, occupying slightly more than half of distance between top of compound eye and lateral ocellus. Antennae dark, A4 apically and A5–12 lightened orange below, A3 equalling A4+5. Ocelloccipital distance equalling width of lateral ocellus. ***Mesosoma***: Scutum and scutellum densely punctured, punctures separated by 1–1.5 puncture diameters, underlying surface of the scutum shagreened anteriorly, elsewhere and on the scutellum shiny. Episternum microreticulate, dull. Lateral faces of the propodeum shagreened, weakly shining. Hind face of propodeum microreticulate and weakly rugose, rugosity outlining the propodeal triangle which is itself rugose and therefore defined by the change in texture between rugosities (Fig. 65). Scutum and scutellum with very short and fine hairs, scarcely longer than width of lateral ocellus. Episternum and propodeum with longer white hair, never exceeding length of the scape. Legs dark, tarsi brown, pubescence brown. Femoral scopa white, tibial scopa dirty brown. Wings hyaline, venation brown, stigma dark brown. Nervulus slightly antefurcal. ***Metasoma***: Tergites dark brown throughout with exception of two lateral black spots on T2 (Fig. 66). Tergites densely, finely, and evenly punctured, punctures separated by one puncture diameter. T2–4 with dense white hair bands that obscure underlying surface, on T2 interrupted medially, on T3–4 complete. T5–6 with thick brown hairs flanking pygidial plate, with a few white hairs laterally. Pygidial plate with slightly raised margin, centrally flat.

**Male.** Unknown.

##### Distribution.

South-western Morocco (Fig. 145b).

##### Floral preferences.

None recorded.

##### Remarks.

Whilst *A. tenebricorpus* is structurally very similar to *A. gafsensis*, the subtle morphological differences suggest that *A. tenebricorpus* is distinct. Moreover, the Souss valley to the Guelmim region is bio-climatically quite distinct to the deserts of southern Tunisia and hosts many unique bee species within Morocco itself, further suggesting a distinct specific identity.

##### Etymology.

The name *tenebricorpus* from *tenebris* (dark) + *corpus* (body) was chosen because of the structural similarities between *A. tenebricorpus* and *A. gafsensis*, but without the same reddish colouration.

#### Andrena (Notandrena) acutidentis

Taxon classificationAnimaliaHymenopteraAndrenidae

Wood sp. nov.

79395A28-0E7C-509D-8C1D-A3FDF70D963D

http://zoobank.org/4F7E28FC-64A4-43D8-AE09-3A7B7E63FA2F

Figures 67–70 , 71–76

##### Material.

***Holotype***: Morocco: Souss-Massa, 10 km SE Ait Baha, 18.iv.1996, 1♀, leg. M. Schwarz. Deposited in the OÖLM. ***Paratypes***: Morocco: Souss-Massa, 10 km SE Ait Baha, 18.iv.1996, 2♂, 2♀, leg. M. Schwarz; 20.iv.1996, 2♂, leg. M. Schwarz; Souss-Massa, 10 km W Tiznit, 6.v.1995, 5♂, 1♀, leg. Mi. Halada, OÖLM; Souss-Massa, 30 km SE Taliouine, 17.iv.1996, 3♀, leg. M. Schwarz, OÖLM; Souss-Massa, Biougra-Tafraout, 13.ii.1987, 1♂, leg H. Teunissen, NMNL. Paratypes are deposited at the OÖLM and NMNL, with a male and female retained in the personal collection of TJW.

##### Diagnosis.

A small Notandrena recognised in the subgenus by the dorsolateral angle of the pronotum with a transverse ridge, the clearly punctured metasoma, and the weakly rugose (not shagreened) propodeal triangle. Because of its small size it can be placed into the *nitidiuscula* group, and it is most similar to *A. fulvicornis* Schenck, 1853 (see also species newly recorded for Morocco below). It differs by the clypeus which has a central, shining, impunctate line (Fig. 68, evenly punctured and shagreened in *A. fulvicornis*) and the shiny scutum and scutellum (Fig. 69, shagreened and dull in *A. fulvicornis*).

The male can easily be recognised as a *Notandrena* because of the greatly enlarged and carinate gena (Fig. 73) and the broadened apex of the gonostyli in combination with a punctured metasoma (Fig. 75). It can be further recognised within the *Notandrena* by the shape of the genitalia which are short and compact (Fig. 76), placing it close to species like *A. chrysosceles*, *A. fulvicornis*, *A. nitidiuscula*, and *A. pallitarsis* (see illustrations in Schmid-Egger and Scheuchl 1997). However, the apices of the gonostyli are produced into points whereas they are rounded in the other species, the clypeus is yellow (Fig. 72, black in species like *A. fulvicornis* and *A. nitidiuscula*), and the apex of sternite 8 is also emarginate. The combination of these characters is unique.

##### Description.

**Figures 67–70.**
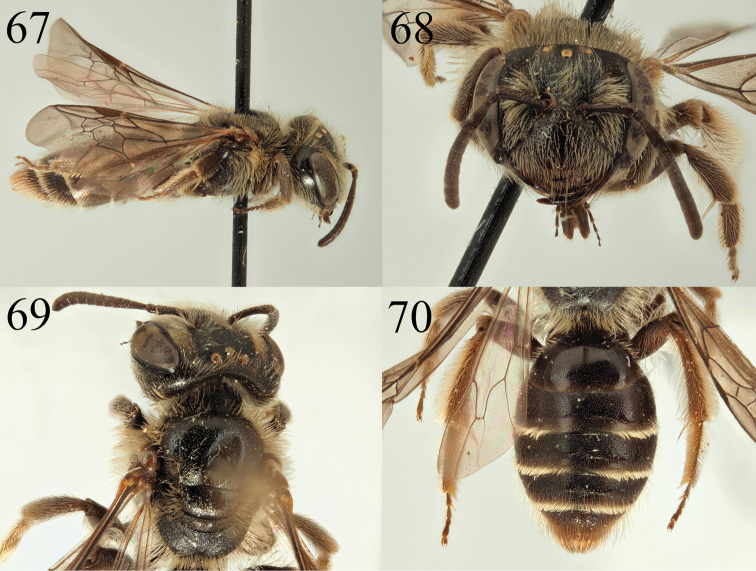
Andrena (Notandrena) acutidentis sp. nov. **67** female profile **68** female face **69** female dorsum **70** female tergites.

**Female**: Body length 8–8.5 mm (Fig. 67). ***Head***: Black, head wider than long (Fig. 68). Clypeus broad, evenly arched, evenly punctured, punctured separated by one puncture diameter with the exception of clear impunctate line in centre equivalent to two puncture diameters. Underlying surface weakly shagreened, shining. Process of labrum trapezoidal, weakly emarginate. Gena broad, 1.2 times wider than width of compound eye, clearly and evenly punctured, punctures separated by one puncture diameter, underlying surface close to the compound eye shining, becoming shagreened towards the hind margin of the vertex, punctures becoming obscure. Gena, vertex, and face with moderate brown hairs, never exceeding the scape in length. Foveae of average width, occupying half distance between top of compound eye and lateral ocellus. Antennae dark, A4 apically and A5–12 lightened orange below, A3 slightly shorter than A4+5+6. Ocelloccipital distance short, ½ width of lateral ocellus. ***Mesosoma***: Scutum and scutellum moderately densely punctured, punctures separated by 1–1.5 puncture diameters, underlying surface weakly shagreened, generally shiny (Fig. 69). Scutum and scutellum with short, fine, brownish hair. Episternum and propodeum microreticulate, dull, propodeal triangle clearly marked by a faint carina, propodeal triangle weakly rugose. Episternum and propodeum with longer brownish-whitish hair, not exceeding length of the scape, propodeal corbiculae well defined. Legs dark, tarsi lightened brown, pubescence yellowish to whitish. Femoral and tibial scopa white. Wings hyaline, venation and stigma dark brown. Nervulus interstitial. ***Metasoma***: Tergites dark, margins slightly lightened yellow to light brown (Fig. 70). T1 moderately punctured, puncture separated by 2–3 puncture diameters, T2–4 more densely punctured, punctures separated by 1–1.5 puncture diameters, underlying surface weakly shagreened, shining. T2–4 with thin hairbands of yellowish-white hairs, on T2+3 broadly interrupted, on T4 complete. T5+6 with a fringe of golden hairs.

**Figures 71–76.**
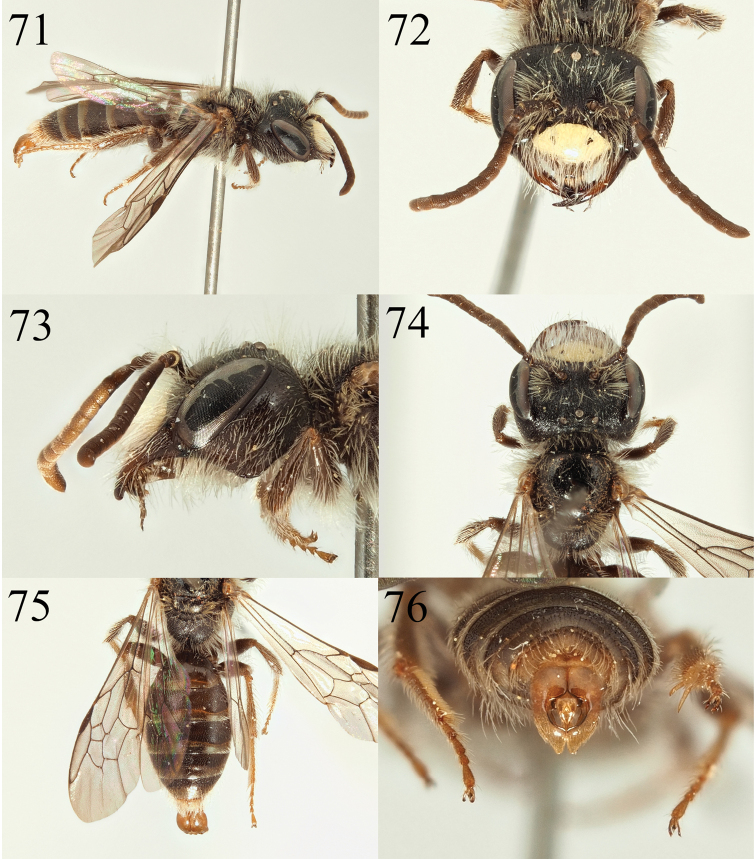
Andrena (Notandrena) acutidentis sp. nov. **71** male profile **72** male face **73** male gena **74** male dorsum **75** male tergites **76** male genitalia.

**Male.** Body length 8 mm (Fig. 71). ***Head***: Black, head wider than long (Fig. 72). Clypeus broad, evenly arched, entirely yellow with exception of two small dark triangles laterally. Process of labrum narrow, longer than broad, emarginate. Gena substantially wider than width of compound eye, angulate with distinct upper and lower corner, carinate (Fig. 73). Genal punctation and sculpturing as in female. Gena, face, vertex, and scape with white hairs, longest equalling scape in length. Antennae dark, A3 apically and A4–13 lightened orange below, A3 equalling A4+5. Ocelloccipital distance short, 2/3 width of lateral ocellus. ***Mesosoma***: Mesosomal sculpturing and pubescence as in the female (Fig. 74). Legs brown, tarsi light brown, pubescence whitish. Wings hyaline, venation and stigma dark brown. Nervulus slightly antefurcal. ***Metasoma***: Tergites brownish, punctation and sculpturing as in the female (Fig. 75). Tergites 2–4 with very thin and faint white hair bands, very broadly interrupted. Sternite 8 apically emarginate and bilobed. Genitalia short and compact (Fig. 76), gonocoxites not forming points, dorsal surface shagreened in a manner reminiscent of *Zonandrena*. Gonostyli forming a point.

##### Distribution.

The Souss valley in south-western Morocco (Fig. 145d).

##### Floral preferences.

None recorded. Other members of the *Notandrena* are associated with Apiaceae (Wood et al. 2020a).

##### Etymology.

The name *acuti* (sharp) + *dentis* (teeth) was chosen because of the male genitalia where the apices of the gonostyli are produced into points in contrast to other members of this group where they are rounded.

#### Andrena (Poecilandrena) nigriclypeus

Taxon classificationAnimaliaHymenopteraAndrenidae

Wood sp. nov.

86C1DF05-B8F9-51E7-9A03-8E7B1226AC9D

http://zoobank.org/53B24FC1-90C7-41A2-8CB2-1AC4E226C2C5

Figures 77–82

##### Material.

***Holotype***: Algeria: Tlemcen, 20 km N de Maghnia, Bab Taza, 34.968N, -1.7622W, 9.iv.1983, 1♂, leg. R. Leys & P. v. d. Hurk. Deposited in the NMNL.

##### Diagnosis.

The subgenus Poecilandrena is poorly defined and has been treated as a wastebasket for species with few apomorphies (Pisanty et al. 2018), and so unsurprisingly it has been found to be strongly polyphyletic, containing at least five clades (Pisanty et al. 2020), and in future revisions, only the *labiata* and *viridescens* groups are likely to remain in the *Poecilandrena*. However, until this point it is desirable to keep superficially similar species together, even if the subgenus itself is clearly polyphyletic, so that they may be dealt with together in future revisions. Based on the criteria outlined by Pisanty et al. (2018), *A. nigriclypeus* can be placed into the *Poecilandrenasensu lato* by the combination of small body size (< 10 mm), red marked abdomen, non-carinate pronotum, mesepisternum weakly areolate with large, spaced shallow punctures, propodeal triangle moderately rugose, genal area not broadened, and sternite eight columnar.

Currently only one species of *Poecilandrenasensu lato* is known from North Africa, the similarly red-marked *A. maximiliani* Scheuchl, 2009 which was described from Tunisia (see Scheuchl 2009 for discussion of previous unconfirmed records of *Poecilandrena* from North Africa). *Andrena nigriclypeus* is similar to *A. maximiliani* as both have male genitalia with pronounced but apically truncate (square-ended) gonocoxal teeth. However, it can be easily separated because the clypeus is black, not yellow, and the gonostyli are completely different, small and narrow and with a shallow emargination in the outer edge before the apex whereas in *A. maximiliani* they are long, broad, and flattened. The only other similar species is *A. paradisaea* Warncke, 1975 from Turkey which has a red abdomen and a black clypeus in the male. However, this species has clearly hooked hind tibial spurs and the genitalia are completely different with a broad-based penis valve, spatula-shaped gonostyli with an evenly rounded outer margin, and pointed gonocoxal teeth (see photograph in Pisanty et al. 2018: fig. 122).

##### Description.

**Female**: Unknown.

**Figures 77–82.**
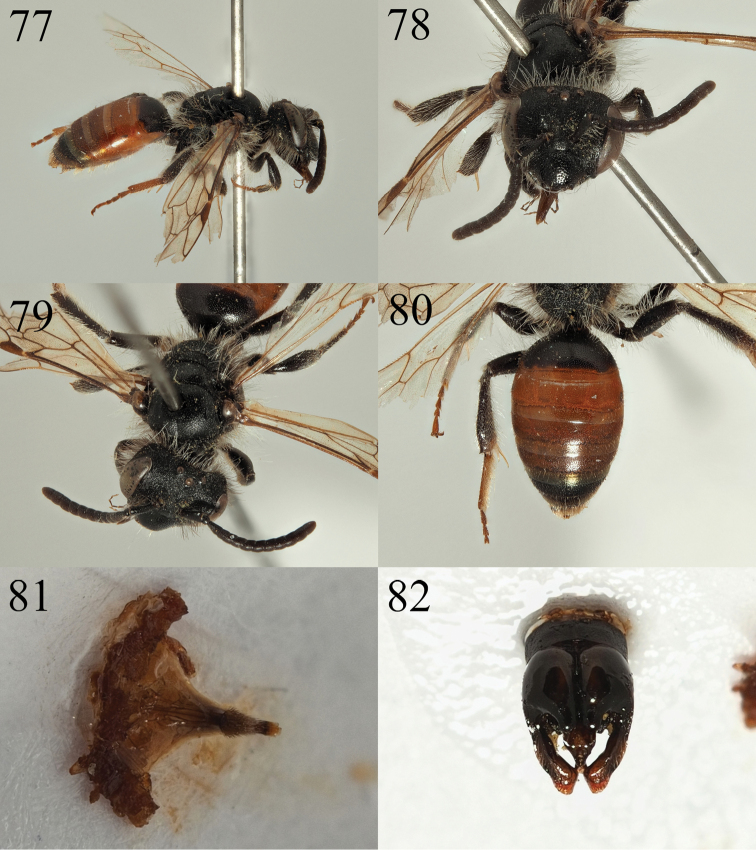
Andrena (Poecilandrena) nigriclypeus sp. nov. **77** male profile **78** male face **79** male dorsum **80** male tergites **81** male sternite eight **82** male genitalia.

**Male.** Body length 8 mm (Fig. 77). ***Head***: Black, slightly broader than long (Fig. 78). Clypeus domed, centrally slightly flattened therefore appearing weakly three-faced. Front 2/3^rds^ weakly shagreened, broadly shiny, basal 1/3 and lateral areas strongly shagreened, dull. Shiny area unevenly punctured, punctures separated by 1–2 puncture diameters, puncture density increases slightly in dull areas, punctures separated by one puncture diameter. Process of labrum rectangular, two times broader than long. Gena and vertex with white hairs, turning to black hairs on gena behind dorsal 1/3 of compound eyes (Fig. 79). Scape with short white hairs, lower paraocular areas and areas around the antennal insertions with mixture of black and white hairs. None of the facial pubescence exceeds scape in length. A1–3 dark, A4–13 dark brown, A3 equalling A4+5. Ocelloccipital distance short, ½ width of lateral ocellus. ***Mesosoma***: Scutum and scutellum densely shagreened, dull, slightly shiny centrally. Surface evenly and weakly punctured, punctures separated by 1–2 puncture diameters. Episternum and propodeum strongly microreticulate, with weak raised reticulation, very weakly shining. Episternum with very shallow wide punctures between raised reticulation. Propodeal triangle well-marked with raised external carina, internal surface with weakly raised longitudinal rugosity. Legs dark, tarsal segments 2–5 on first two pairs of legs and all segments including the basitarsi on hind legs lightened dark red-orange (Fig. 77). Wings hyaline, venation amber, nervulus interstitial. ***Metasoma***: T1 predominantly dark, red-marked only on apical margin, T2–4 red, T5–6 black (Fig. 80). T2–5 with apical margins lightened yellow, slightly hyaline apically. All tergites densely and uniformly punctate, punctures separated by 0.5 puncture diameters, underlying surface finely shagreened, weakly shining. T6+7 with short golden hairs. S8 arched, centrally at vertical apex of the arch with a patch of dense yellowish hairs, remaining part of relatively hairless sternite narrowly projecting beyond (Fig. 81). Genitalia simple, gonocoxites with pronounced but apically truncate and square-ended teeth (Fig. 82). Penis valve triangular basally with slightly raised winged margins. Gonostyli narrow, apically strongly truncate with a shallow emargination in outer margin (Fig. 82).

##### Distribution.

North-western Algeria close to the Moroccan border (Fig. 145d).

##### Floral preferences.

None recorded.

##### Etymology.

The name *nigri* (black) + *clypeus* (clypeus) was chosen because of the entirely black male clypeus, which is unusual within small red-marked *Andrena* species.

#### Andrena (Poliandrena) breviceps

Taxon classificationAnimaliaHymenopteraAndrenidae

Wood sp. nov.

2CA14BAE-677E-5F5D-9D17-49202FF5D7F8

http://zoobank.org/8F5F1214-8FDB-404D-BB5C-E9C0F7E8031B

Figures 83–86

##### Material.

***Holotype***: Morocco: Drâa-Tafilalet, 10 km N Erfoud, 10.iv.1995, 1♀, leg. Ma. Halada. Deposited in the OÖLM. ***Paratypes***: Morocco: Drâa-Tafilalet, 10 km N Erfoud, 10.iv.1995, 2♀, leg. Ma. Halada, OÖLM; Drâa-Tafilalet, 20 km E Agdz, 20.iv.1995, 2♀, leg. Mi. Halada, OÖLM; Drâa-Tafilalet, Tagounite, 60 km S Zagora, 23.iv.1995, 1♀, leg. Ma. Halada, OÖLM. Paratypes are deposited at the OÖLM with a female retained in the personal collection of TJW.

##### Diagnosis.

The subgenus Poliandrena Warncke, 1968 is currently unsatisfactorily defined (females keying out in three places in Warncke’s 1968a key) and will probably be broken up in the future as it has been shown to be strongly polyphyletic (Pisanty et al. 2020). It predominantly contains Mediterranean species that have heads that are short and broad (Fig. 84), short facial foveae, strongly punctate metasomas (Fig. 86), and often show a propodeal triangle that is defined by an external carina and internal rugosity (but not honeycomb-areolate, Warncke 1968a). As such, members of the *Poliandrena* are often recognised by their similarity to each other rather than by a single character *per se*, in other words a wastebasket taxon.

*Andrena breviceps* is small relative to the rest of the *Poliandrena*, comparable in size to *A. marsae* Schmiedeknecht, 1900 and *A. laurivora* Warncke, 1974, two of the smallest *Poliandrena*, but *A. breviceps* can easily be separated from them by the colour of the tergites which are dark brown (red in *A. marsae*, dark metallic green-blue in *A. laurivora*) and the sculpturing of the scutum which is shiny and (relatively within the *Poliandrena*) sparsely punctate, punctures separated by 2–3 puncture diameters (Fig. 75, separated by less than ½ a puncture diameter in *A. marsae* and by one puncture diameter in *A. laurivora*, underlying integument shagreened, with a metallic glint). It is also similar to the larger *A. relata* Warncke, 1967 which is newly recorded for Morocco (see below) because of their similar dark tergites and general appearance. However, it can also be separated using the same scutal punctation character (in *A. relata* punctures dense laterally, centrally separated by at most two puncture diameters) and also by the clypeus where *A. breviceps* has a central longitudinal impunctate line that is not present in *A. relata*. Overall, the shiny and relatively sparsely punctate scutum (Fig. 75) in combination with its small size should allow separation from other *Poliandrena* from North Africa.

##### Description.

**Figures 83–86.**
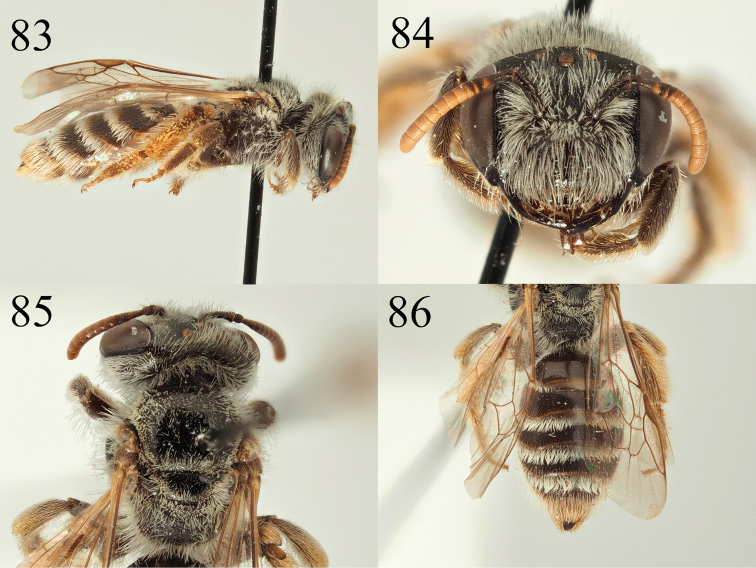
Andrena (Poliandrena) breviceps sp. nov. **83** female profile **84** female head **85** female dorsum **86** female tergites.

**Female**: Body length 8.5–9 mm (Fig. 83). ***Head***: Black, clearly wider than long (Fig. 84). Clypeus broad, arched, with large punctures, punctures separated by one puncture diameter except for a central slightly raised impunctate line, underlying surface uneven, slightly but irregularly raised between punctures, shining. Process of labrum broadly trapezoidal, fore margin weakly emarginate. Gena as wide as width of compound eye. Gena, vertex, face, and scape with moderately dense white hairs, longest of these not exceeding length of the scape. Foveae moderately broad, occupying 2/3 of distance between the of compound eye and lateral ocellus, of normal length, not extending below the level of the antennal insertions. Antennae bright, scape and pedicel dark, A3 apically marked with orange, A4–12 predominantly orange, A3 exceeding A4+5, shorter than A4+5+6. Ocelloccipital distance short, ½ width of lateral ocellus. ***Mesosoma***: Scutum and scutellum moderately punctured, punctures separated by 1–2 puncture diameters, underlying surface shiny, very weakly shagreened laterally and anteriorly (Fig. 85). Margins of scutum and scutellum with short, dense whitish hairs, these extending only very sparsely onto the disc. Episternum and propodeum microreticulate, dull, propodeum with weak rugosity, propodeal triangle defined with small but clear slightly raised carina, propodeal triangle with sparse and weak rugosity centrally. Episternum and propodeum with white hairs, longest not exceeding length of the scape. Legs dark, tarsi lightened brown, pubescence whitish to brownish. Femoral and tibial scopa white. Wings hyaline, venation and stigma brown. Nervulus antefurcal. ***Metasoma***: Tergites brownish, margins slightly depressed, lightened white to yellow, apically translucent (Fig. 86). Tergites densely, evenly, and finely punctate, punctures separated by one puncture diameter, underlying surface very weakly shagreened, shining. T1 with two small lateral hair patches of white hair on apical margin, T2–4 apically with complete white hair bands obscuring underlying surface. T5–6 with golden hairs flanking pygidial plate. Pygidial plate rounded triangular, flat, without raised margin.

**Male.** Unknown.

##### Distribution.

The eastern Moroccan desert in the province of Drâa-Tafilalet (Fig. 145c).

##### Floral preferences.

None recorded.

##### Etymology.

The name *brevi* (short) + *ceps* (head) was chosen because of the particularly short and wide head of this species, even within the *Poliandrena*.

#### Andrena (Poliandrena) farinosoides

Taxon classificationAnimaliaHymenopteraAndrenidae

Wood sp. nov.

6E8256F9-2EAB-5164-A46A-9D615796050F

http://zoobank.org/184F4B1C-A3C6-48A8-96D6-560CC9A9379A

Figures 87–94 , 95–98

##### Material.

***Holotype***: Morocco: Oriental, 40 km S Guercif, 15–17.v.1995, 1♀, leg. Ma. Halada. Deposited in the OÖLM. ***Paratypes***: Morocco: Oriental, 40 km S Guercif, 15–17.v.1995, 2♂, 24♀, leg. Ma. Halada, OÖLM; Drâa-Tafilalet, 30 km E Midelt, 13.v.1995, 1♀, leg. Mi. Halada, OÖLM; Drâa-Tafilalet, 10 km N Rich, 2♀, leg. Mi. Halada, OÖLM. Paratypes are deposited at the OÖLM with three females retained in the personal collection of TJW.

##### Diagnosis.

*Andrena farinosoides* can also be placed in the *Poliandrena* because of its short and broad head (Fig. 88), short facial foveae, strongly punctate metasoma (Fig. 91), and propodeal triangle marked by a carina and internal rugosity (but not honeycomb-areolate). It can be recognised within the *Poliandrena* as very similar to *A. farinosa* Pérez, 1895 from Spain and France (Gusenleitner and Schwarz 2002) but the two species differ in the pubescence of the abdomen and the scutal punctation. In addition to thick hair bands on the tergal margins (Figs 90, 92), *A. farinosa* also has short hairs on the tergal discs forming a sparse velvety pubescence when viewed laterally (Fig. 94, not as dense as in *A. eddaensis* or *A. decaocta*). In contrast, *A. farinosoides* has only a few short hairs on the tergal discs, not forming a velvety pubescence (Fig. 93). In addition, the scutum of *A. farinosoides* is clearly less densely punctate, with punctures separated by one puncture diameter, punctures denser and almost confluent in *A. farinosa*.

Diagnosis of the male is more difficult, but both *A. farinosa* and *A. farinosoides* are in the group with dark, densely punctate tergites and black (not yellow) clypei with an upturned fore margin (Fig. 96). The male of *A. farinosoides* is then less densely and more finely punctate on the scutum (punctures separated by one puncture diameter) whereas *A. farinosa* has larger punctures that are separated by less than a puncture diameter (Fig. 97).

##### Description.

**Figures 87–94.**
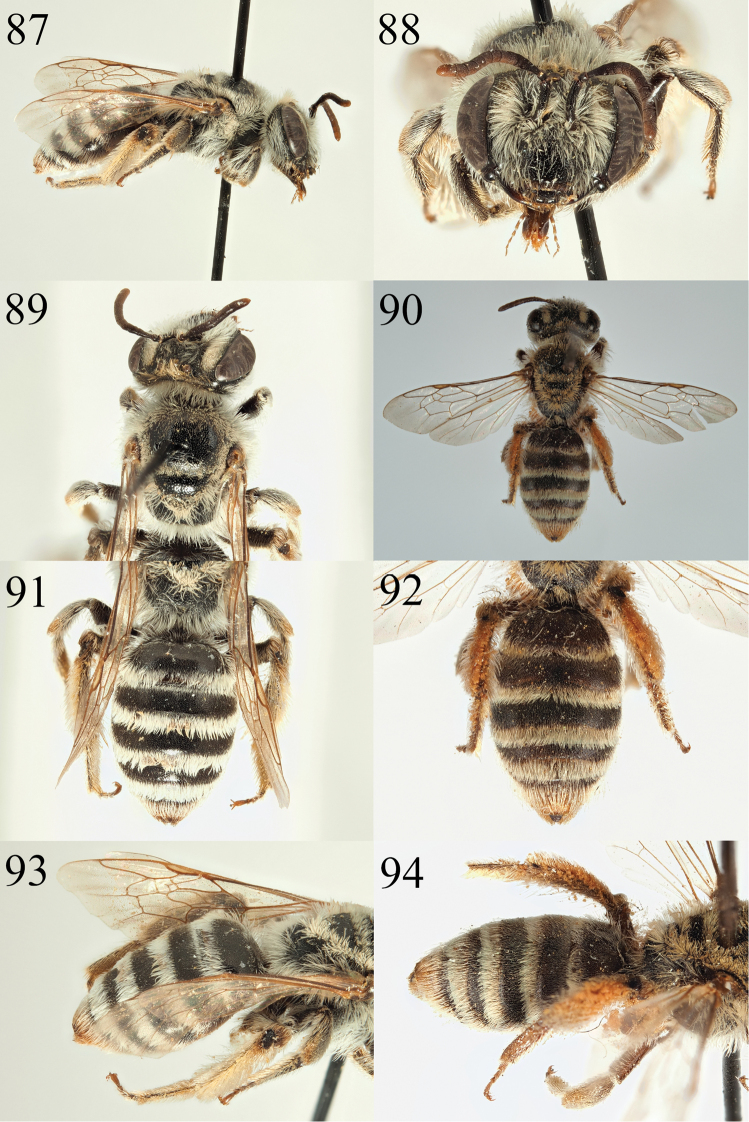
Andrena (Poliandrena) farinosoides sp. nov. **87** female profile **88** female face **89** female dorsum **90** female tergites **91** female tergites in lateral view. Andrena (Poliandrena) farinosa Pérez, 1895 (paralectotype) **92** female dorsum **93** female tergites **94** female tergites in lateral view.

**Female**: Body length 8.5–9.5 mm (Fig. 87). ***Head***: Black, clearly wider than long (Fig. 88). Clypeus broad, slightly arched, densely and evenly punctured, punctures separated by ½ a puncture diameter. Process of labrum broad, trapezoidal, twice as wide as long, fore margin compressed and slightly upturned, surface with weak transverse striations. Gena slightly narrower than width of compound eye. Gena, face, and scape with dense white hairs, longest not achieving ¾ of length of the scape, hairs on the vertex of similar length but becoming yellowish. Foveae normal, occupying half of distance between top of compound eye and lateral ocellus. Antennae dark, A5–12 lightened orange below, A3 long, slightly shorter than A4+5+6. Ocelloccipital distance short, less than 1/3 width of lateral ocellus. ***Mesosoma***: Scutum and scutellum densely punctured, punctures separated by one puncture diameter, underlying surface smooth and shiny (Fig. 89). Margins of scutum and scutellum with short whitish-brownish hairs, densest on the margins and becoming sparser as hairs move into centre of the disc. Episternum and propodeum with weak rugosity, dull, propodeal triangle clearly defined by raised carina, propodeal triangle itself rugose with weak longitudinal carinae. Episternum and propodeum with white hairs, not exceeding ¾ length of scape. Legs dark, tarsi lightened brown, pubescence light brown to whitish. Femoral and tibial scopa white. Wings hyaline, venation and stigma light brown. Nervulus interstitial. ***Metasoma***: Tergites dark, margins depressed and slightly lightened to brown (Fig. 91). Tergites densely and evenly punctured, punctures separated by ½ puncture diameter. T1–4 with thick white hair bands that exceed length of margins, completely obscuring underlying surface, all bands complete in fresh individuals. T5 and T6 with golden hairs flanking pygidial plate, white haired laterally. Pygidial plate rounded triangular, without raised margin, very subtly domed centrally.

**Male.** Body length 7 mm (Fig. 85). ***Head***: Black, clearly wider than long (Fig. 96). Clypeus broadly flattened, with clear upturned fore margin. Clypeus densely and evenly punctured as in female. Gena thickened, slightly wider than the width of compound eye. Gena, face, vertex, and scape with white hairs, longest equalling length of the scape. Antennae dark, A4–13 lightened brown below, A3 slightly shorter than A4+5. Ocelloccipital distance short, ½ width of lateral ocellus. ***Mesosoma***: Similar to female, scutum and scutellum evenly punctured, punctures separated by one puncture diameter, underlying surface shiny, contrasting with the episternum and propodeum that are reticulate, dull (Fig. 97). Legs dark, tarsi lightened brown, pubescence white. Wings hyaline, venation and stigma light brown. Nervulus interstitial. ***Metasoma***: Similar to female, tergites dark, margins lightened yellow to brown, apically whitish translucent (Fig. 98). Tergites densely and evenly punctate, punctures separated by one puncture diameter.

##### Distribution.

The eastern Moroccan desert in the provinces of Oriental and Drâa-Tafilalet (Fig. 145d).

##### Floral preferences.

None recorded.

##### Etymology.

Given the similarity to *A. farinosa*, the name *A. farinosoides* (*farinosa* + *oides*, form or likeness) was chosen to illustrate this close link.

##### Other material examined.

(*Andrena farinosa*): Spain: Barcelona, [no date], 1♀, designated paratype [technically paralectotype] by Warncke, Warncke Colln., OÖLM (illustrated Figs 90, 92, 94); 80 km SW Valencia, Muela de Cortes reserve, 14.v.2003, 4♂, 13♀, leg. J. Halada, OÖLM; Lleida, Granadella, 450 m, 23.v.1983, 2♂, 1♀, leg. H. Teunissen, NMNL; Maella, 23.v.1983, 1♂, leg. H. Teunissen, NMNL; Murcia, Pto. de Jumilla, 19.v.2003, 1♀, leg. J. Halada, OÖLM; Murcia, Sierra de Españula, 11.v.2003, 5♂, 3♀, leg. J. Halada, OÖLM; Taragona, Bellaguarda, 683 m, 1.vi.2019, 1♀, leg. W. Klein, NMNL; Zaragoza, Codos, 5.vi.1985, 2♂, leg. H. Teunissen, NMNL.

**Figures 95–98.**
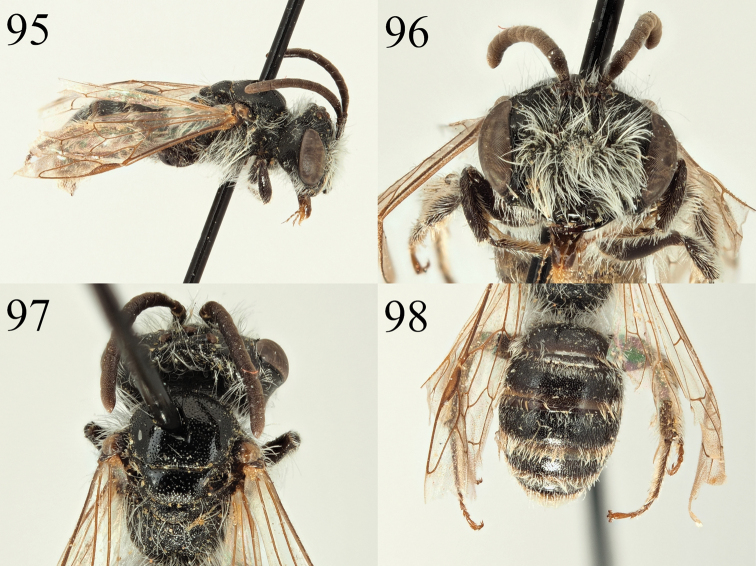
Andrena (Poliandrena) farinosoides sp. nov. **95** male profile **96** male head **97** male dorsum **98** male tergites.

### Additional taxonomic changes and updates

#### Andrena (Aciandrena) fulica

Taxon classificationAnimaliaHymenopteraAndrenidae

Warncke, 1974

C9F37F92-B810-545C-A13F-F53198D0A732

Figures 99–104

Andrena (Aciandrena) astrella ssp. fulica Warncke, 1974: p44
Andrena (Aciandrena) astrella Warncke, 1975: 305. syn. nov.

##### Material examined, males only, black faced and therefore nominally *A. fulica*.

**Figures 99–104.**
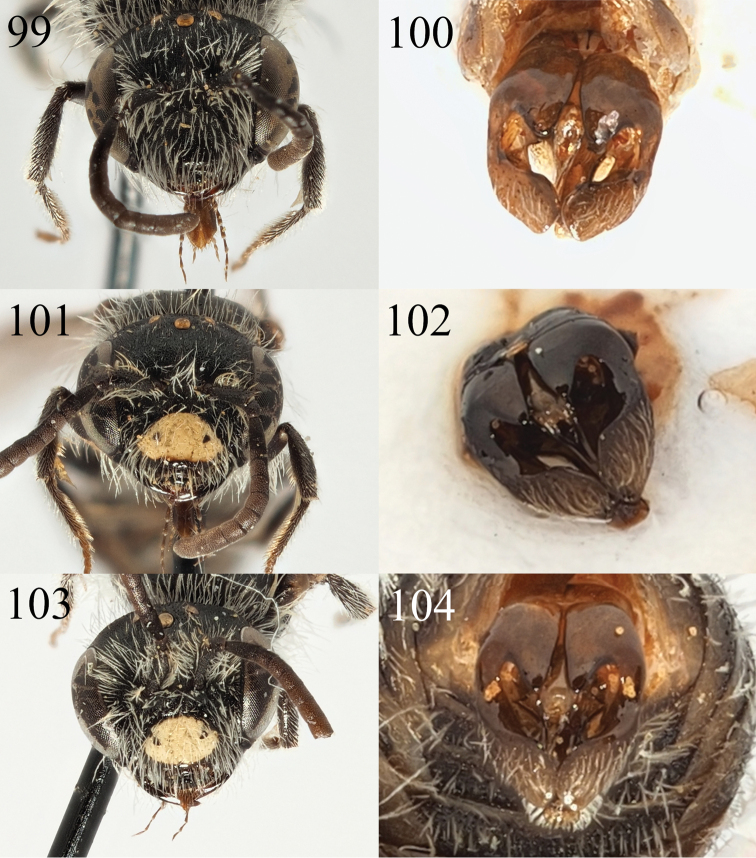
Andrena (Aciandrena) fulica Warncke, 1974 **99** male face **100** male genitalia. Andrena (Aciandrena) fulica astrella Warncke, 1975 (Morocco) **101** male face **102** male genitalia (Spain) **103** male face **104** male genitalia.

Tunisia: Tunis, 1898, 1♂, leg. O. Schmiedeknecht (paratype *A. fulica*), OÖLM; Morocco: Fès-Meknès, Ifrane environs, 9.v.1997, 1♂, leg. K. Deneš, OÖLM (illustrated Figs 99, 100); Spain: Benidorm, 20.iv.1982, 1♂, NMNL.

##### Material examined, males only, yellow face and therefore nominally *A. astrella*.

Morocco: Fès-Meknès, Ifrane environs, 9.v.1997, 1♂, leg. K. Deneš, OÖLM (illustrated Figs 101, 102); Portugal: Algarve, Altura, near Monte Gordo, 24.iv.2016, 1♂, leg. Wood, TJW Colln.; Algarve, Pêra, Praia Grande, 2.iv.2015, 2♂, leg. Wood, TJW Colln.; Spain: Rivas [Madrid], 1♂, leg. Dusmet (paratype *A. astrella*), OÖLM; 40 km W Malaga, Yunquera, 800 m, 29.iv.2003, 2♂, leg. J. Halada, OÖLM; 50 km W Almería, Berja, 21.iv.2003, 10♂, leg. J. Halada, OÖLM; Alicante, Elche, 23.iv.1982, 1♂, NMNL; Altea, 10 km N Benidorm, 15.iv.1982, 1♂, NMNL; Benidorm, 20.iv.1982, 1♂, NMNL; Ávila, Hoyos del Espino, 1400 m, 19.v.1995, 1♂, leg. H. & J.E. Wiering, NMNL; Ávila, Hoyocasero, 1350 m, 2 km W, 10.v.1995, 1♂, leg. H. & J.E. Wiering, NMNL; 80 km SW Valencia, Muela de Cortes reserve, 14.v.2003, 2♂, leg. J. Halada, OÖLM; E-Sierra Nevada, near Alboloduy, 7.v.2003, 3♂, leg. J. Halada, OÖLM; Sierra Alhamilla, 5 km E Nijar, 20.iv.2003, 1♂, leg. J. Halada, OÖLM; Sierra Alhamilla, Lucainena, 25.iv.2003, 10♂, leg. J. Halada, OÖLM; Sierra de Nevada, Ohanes env., 5.v.2003, 5♂, leg. J. Halada, OÖLM; S-Sierra Nevada, env. Lanjaron, 4.v.2003, 5♂, leg. J. Halada, OÖLM (illustrated Figs 103, 104).

##### Distribution and remarks.

*Andrena fulica* (Morocco, Algeria, and Tunisia) and *A. astrella* (Spain and Portugal) were both described by Warncke, with *A. fulica* described as A. astrella ssp. fulica (Warncke, 1974) despite being formally published before *A. astrella* (Warncke 1975b), this problem arising due to differences in publishing speed between the two journals, with the paper on Iberian *Andrena* originally submitted in 1971. The most recent global treatment listed them as distinct (Gusenleitner and Schwarz 2002).

The two species can be rapidly recognised within the *Aciandrena* because they have punctured tergites with the punctures extending onto the tergal margins, a character that is unique within this subgenus. According to Warncke (1974), *Andrena fulica* is characterised as having finer tergal punctation and, importantly, a black clypeus in the male sex (Fig. 99), whereas in *A. astrella* the male clypeus is yellow (Figs 101, 103). The description of *A. fulica* by Warncke (1974) is extremely short, just two sentences for the female and two short sentences for the male which read: ‘♂ wie beim ♀ feiner punktiert. Clypeus dunkel gefärbt!’.

Inspection of *Aciandrena* males from Ifrane in Morocco (9.v.1997) revealed the two colour forms in sympatry, though unfortunately only two specimens in total (one *A. fulica*, one *A. astrella*) were collected during this sampling making an assessment of variation impossible. Additionally, specimens from Benidorm in Spain (20.iv.1982) also showed the two colour forms in sympatry. Across all Iberian material studied, 46/47 (97.8%) males showed a consistently yellow clypeus. Inspection of the genitalia showed no obvious differences between the two Ifrane specimens (Figs 100, 102), and neither specimen shows obvious differences from the genitalia of Spanish specimens (Fig. 104).

*Aciandrena* do not always show consistent clypeal colouration. In the recently described A. (Aciandrena) abruptifovea Wood, 2020, a series of six males from the same type locality site collected on the same day vary considerably in colouration from a clypeus with 80% yellow coverage, to 40%, to entirely black (Wood et al. 2020b). Given the extremely similar genitalia and the fact that both colour forms can be found together in both Iberia and North Africa, we synonymise *A. astrella* with *A. fulica*, with *A. fulica* taking priority as senior synonym as it was published first.

#### Andrena (Nobandrena) ounifa

Taxon classificationAnimaliaHymenopteraAndrenidae

Warncke, 1974

CEA80990-0F6B-5BB4-AFC4-652ADCC51C54

Figs 105–112

##### Material examined.

Morocco: Drâa-Tafilalet, Errachidia, 11.iv.1995, 2♀, leg. Ma. Halada, one female deposited in the OÖLM, with one female retained in the personal collection of TJW.

##### Distribution and remarks.

Previously known only from the type locality in the western Algerian part of the Sahara Desert. The specimens from Errachidia are approximately 350 kilometres to the west. They agree with the male in size, the flattened and shiny clypeus (Figs 106, 108), the length of the propodeum (Figs 107, 111), and general structural characters. The female differs most strongly from classical *Nobandrena* (see Warncke 1968a) in the shape of the foveae which are long and very narrow, narrower than the width of an antenna (Fig. 106) and on the tergites it lacks the impressed centre line on T(2)3–4. In common with *A. iliaca* which was also placed in the *Nobandrena* by Warncke, it has a long propodeum with a flat, granular dorsal surface. Molecular work places *A. iliaca* within the *Fuscandrena* (Pisanty et al. 2020), and it may be moved there in future, but without molecular data this move is premature, though it clearly does not belong in the *Nobandrena*. The holotype male is illustrated here for comparison (Figs 109–112). A single pollen load contained pure *Raphanus*-type pollen (Brassicaceae).

##### Description.

**Figures 105–112.**
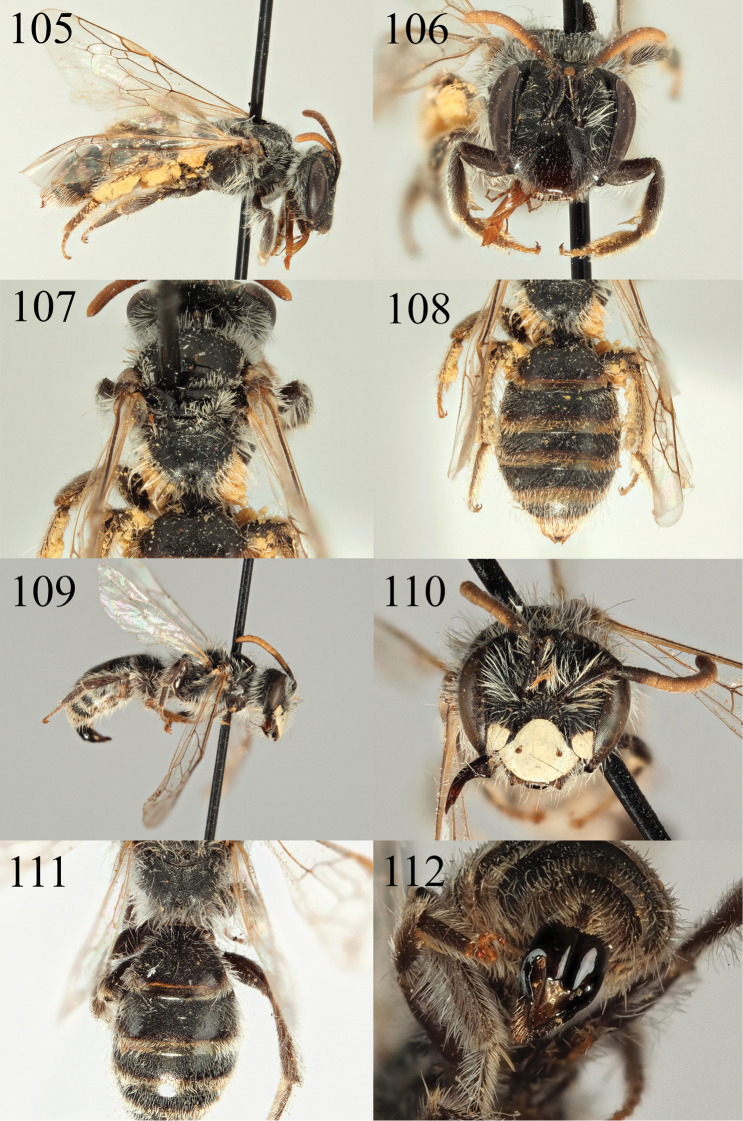
Andrena (Nobandrena) ounifa Warncke, 1974 **105** female profile **106** female face **107** female propodeum **108** female tergites **109** male (holotype) profile **110** male face **111** male tergites **112** male genitalia.

**Female**: Body length 7.5 mm (Fig. 105). ***Head***: Black, clearly longer than wide (Fig. 106). Clypeus strongly flattened, strongly shining, weakly shagreened only on margins, moderately punctured, punctures separated by 2–3 puncture diameters. Process of labrum weakly trapezoidal, almost triangular, broad, twice as wide as long. Gena slightly narrower than width of compound eye with sparse, short white hair extending to vertex. Foveae long, narrow, narrower than width of scape, separated from inner margin of compound eye by less than their own width. Antenna dark, A4–12 extensively lightened orange below, A7–12 almost entirely orange, A3 slightly exceeding A4+5 in length. Ocelloccipital distance extremely short, linear, almost non-existent. ***Mesosoma***: Scutum strongly shagreened, dull to weakly shining centrally, strongly contrasting with shiny scutellum. Scutum and scutellum moderately punctured, punctures separated by 2–3 puncture diameters, with short whitish-brown pubescence. Episternum and propodeum strongly shagreened, dull. Dorsal area of propodeum longer than scutellum, propodeal triangle indicated by an increase in granular size of shagreenation (Fig. 107). Episternum and propodeum with white hair, longest attaining ¾ of length of the scape. Legs dark, tarsi lightened brown, pubescence whitish-brownish. Femoral and tibial scopa white. Wings hyaline, venation brown, stigma light brown. Nervulus interstitial. ***Metasoma***: Tergites dark, margins clearly lightened yellow to dark orange, apically partly translucent (Fig. 108). Tergal discs microreticulate, dull. Tergal discs and margins with sparse white hair, T5 apically and T6 with short golden hairs flanking the pygidial plate. T5 basally with long white hairs, these overlaying but not obscuring the apical golden hairs. Pygidial plate narrow, centrally with a longitudinal slightly raised area.

##### Other material examined.

Algeria: Beni Ounif, 6.iii.[year unknown], 1♂, leg. Weber, OÖLM (holotype), illustrated Figs 109–112.

#### Andrena (Poliandrena) guichardi

Taxon classificationAnimaliaHymenopteraAndrenidae

Warncke, 1980

9043B022-FBCC-57B1-9FFC-20E29104FC0F

Figures 113–116

##### Material examined.

Morocco: Guelmim-Oued Noun, 15–16.iv.1995, 3♂, 1♀, leg. Ma. Halada, OÖLM; Souss-Massa, 10 km S Taroudant, 12.iv.1995, 1♀, leg. Ma. Halada, allotype male and one other male deposited in the OÖLM, with one male and one female retained in the personal collection of TJW.

##### Distribution and remarks.

Known only from south-western Morocco (Warncke 1980). Gusenleitner and Schwarz (2002) provided comments on the appearance of the male but this was not a formal description and so one is provided here. *Andrena guichardi* can be easily recognised within the *Poliandrena* by the extremely shiny and very sparsely punctate scutum (Fig. 115).

##### Description.

**Figures 113–116.**
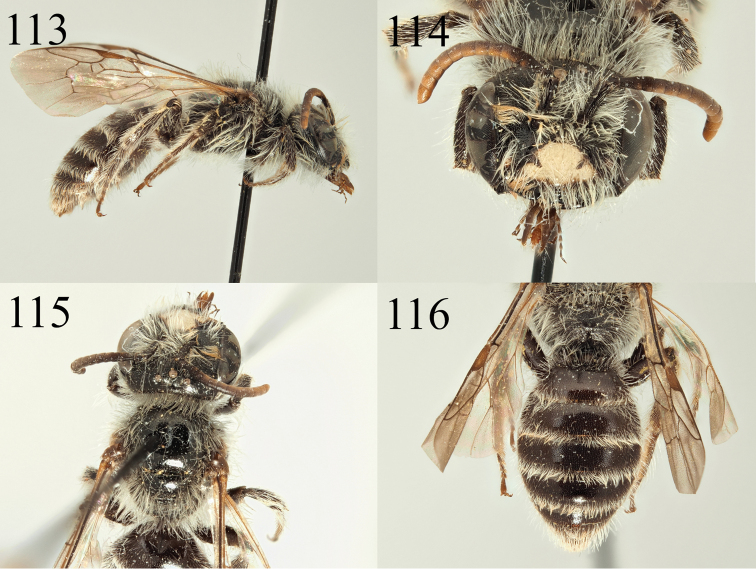
Andrena (Poliandrena) guichardi Warncke, 1980 **113** male profile **114** male face **115** male dorsum **116** male tergites.

**Male**: Body length 9 mm (Fig. 113). ***Head***: Black, clearly wider than long. Clypeus slightly arched, predominantly yellow but with two dark triangular marks intruding laterally (Fig. 114). Underlying surface smooth and shiny, densely punctured, punctures separated by one puncture diameter except centrally where there is a small impunctate circular area. Process of labrum narrow, weakly emarginate, shiny. Gena slightly wider than the width of a compound eye. Gena, face, vertex, and scape with long white hairs, equalling length of the scape. Antennae dark, A3 apically and A4–12 completely lightened orange below, A3 exceeding A4, shorter than A4+5. Ocelloccipital distance broad, two times wides than width of lateral ocellus. Surface of galea shagreened, dull. ***Mesosoma***: Scutum and scutellum sparsely punctate, punctures separated by 2–4 puncture diameters, underlying surface very smooth and shiny (Fig. 115). Episternum weakly reticulate, underlying surface weakly shining. Propodeum microreticulate, dull, propodeal triangle marked by small but clearly defined slightly raised carina, internal structure weakly and finely rugose. Scutum, scutellum, episternum, and propodeum with long white hairs, these often exceeding the length of the scape. Legs dark, tarsi lightened brown, pubescence white. Wings hyaline, venation and stigma brown, nervulus antefurcal. ***Metasoma***: Tergites dark brownish, margins lightened whitish, apically translucent (Fig. 116). Tergal discs very weakly reticulate, shining, tergites densely and finely punctured, punctures separated by one puncture diameter. T1–5 with loose white hair bands, on T1 interrupted, T2–5 complete. T6–7 centrally with golden fringe of hairs. S2–5 with apical fringes of white hair.

##### Other material examined.

Morocco: Sidi-Ifni, within 100 m of the sea, 31.iii.1974, 1♀, leg. K.M. Guichard & G.R. Else, OÖLM**(*paratype*)**.

#### Andrena (Truncandrena) alchata

Taxon classificationAnimaliaHymenopteraAndrenidae

Warncke, 1974

61C4A24B-44BB-5CA9-99E2-F33A0C988DE9

##### Material examined.

*Andrena alchata*: Algeria: Maison Carrée, Alger, 1♂, leg. Dr. J. Bequaert, OÖLM**(*holotype*)**; *Andrena doursana agadira*: Morocco: S-Marokko, Agadir, 20.ii.1977, 1♀, OÖLM**(*holotype*)**; *Andrena doursana citreola* Warncke, 1975: Spain: Vaciamadrid, 25.v.1919, 1♀, leg. Dusmet, OÖLM**(*holotype*)**; Morocco: Azrou Ras el Ma, 30.iii.1923, 1♀, leg. Schulthess, OÖLM**(*paratype*)**; Rez Dj. Zalagh, 25.iii.1923, 1♂, leg. Schulthess, OÖLM**(*paratype*)**.

##### New material.

*Andrena alchata*: Morocco: Fès-Meknès, Laanoucer, 33.6167, -4.7489, 2.v.2018, 1♂, 3♀, leg P. Lhomme & A. Sentil; 33.6708, -4.8527, 2–3.v.2018, 1♀, white pan trap, leg P. Lhomme & A. Sentil; 33.6699, -4.8673, 10–11.v.2018, 1♂, 1♀, yellow pan trap, leg P. Lhomme & O. Ihsane; 33.7099, -4.8431, 15–16.v.2018, 1♀, white pan trap, leg. P. Lhomme & O. Ihsane; 7099, -4.8431, 15–16.v.2018, 1♀, white pan trap, leg. P. Lhomme; Casablanca-Settat, Oueled Sghir, 32.8230, -7.6421, 23.ii.2018, 1♂, 1♀, A. Sentil & I.E. Abdouni, all UMONS.

##### Distribution.

*Andrena alchata* is known from Morocco and Algeria and was only described from the male sex (Warncke 1974); the subspecies *A. doursana agadira* was known only from south-western Morocco and was described only from the female sex (Warncke 1980), though the presence of putative males was noted.

##### Remarks.

Sampling at both Laanoucer and Oueled Sghir in northern Morocco resulted in the capture of males of *A. alchata* and females corresponding to *A. doursana agadira* on the wing at the same time. Other *Truncandrena* species were present, with females of *Andrena ferrugineicrus* Dours, 1872 and *A. schmiedekneckti* Magretti, 1883 captured at Laanoucer and males and females of *Andrena varia* Pérez, 1895 at Oueled Sghir. However, no males or females of *A. doursana citreola* Warncke, 1975, the form occurring in this part of Morocco, were recorded. This situation raised the possibility that *A. alchata* and *A. doursana agadira* are actually synonymous.

**Figures 117–124.**
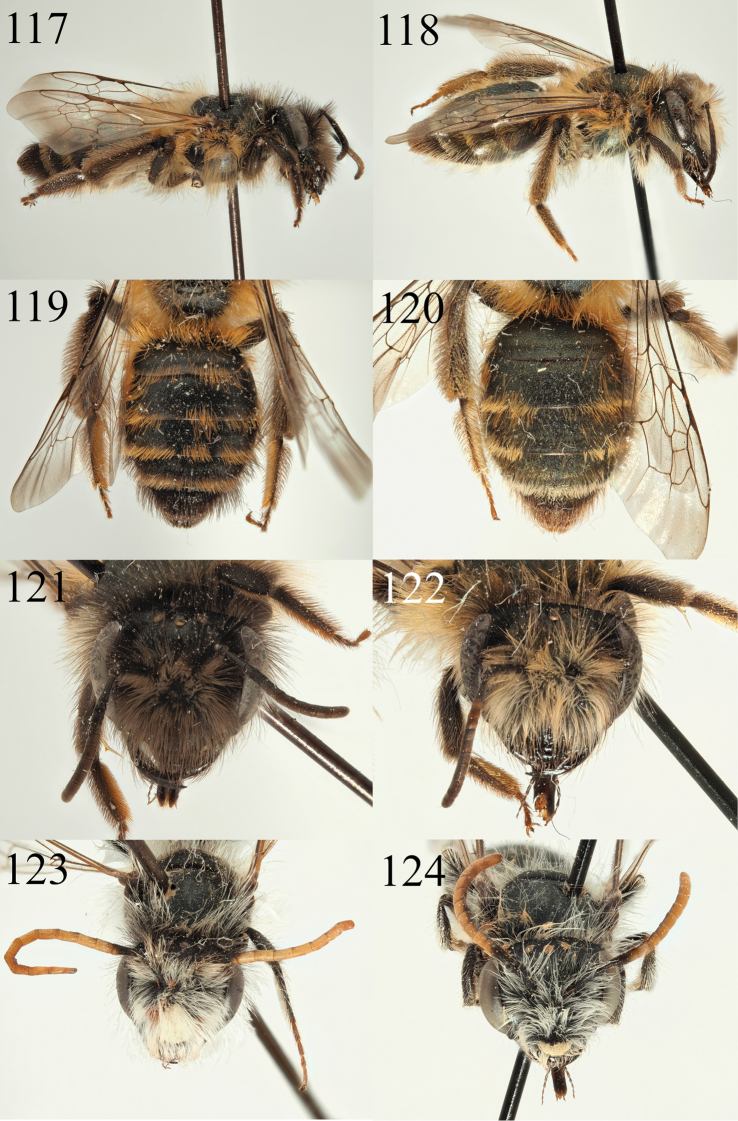
Andrena (Truncandrena) alchata Warncke, 1974 **117** female profile **119** female tergites **121** female face **123** male face. Andrena (Truncandrena) doursana citreola Warncke, 1975 **118** female profile **120** female tergites **122** female face **124** male face.

Captured females are darker than *A. d. citreola* (compare Figs 117, 118) and can be separated by the colouration of the hairs of T5–6 flanking the pygidial plate which are dark brown (Fig. 119) not light brown as in *A. d. citreola* (Fig. 120). The contrast of these hairs against the abdomen is greater in *A. d. citreola* as the underlying integument is lighter and weakly metallic green (see also Fig. 118) with overlying pale hair bands, whereas in captured females the integument is dark and the hair bands are darker (Fig. 119). The colouration of the hairs on the frons and along the inner margin of the compound eye are also important; these are a mixture of brown and black in captured females (Fig. 121), and white to light brown in *A. d. citreola* s.s. (Fig. 122). In these characters, captured females match *A. d. agadira* perfectly (Warncke 1980).

Warncke did not describe male of *A. d. agadira*, commenting that they were indistinguishable from other *A. doursana* subspecies. In contrast, males of *A. alchata* are easy to distinguish as the white markings on the face of *A. alchata* are much more extensive, covering the clypeus and the lower paraocular areas (Fig. 123) whereas in *A. d. citreola* the single white marking is small and is restricted to the very tip of the clypeus which forms a clear raised protrusion (Fig. 124). There are also genitalia differences, in *A. alchata* the outer margin of the gonostyli is curved inwards (evenly rounded in *A. d. citreola*), and the penis valve is comparatively wider.

A female corresponding to *A. d. agadira* and a male *A. alchata* were selected from the same site (Laanoucer, 2.v.2018) for molecular investigation. A *cox1* fragment of 263 base pairs was obtained after sequencing. A complete homology between the sequences of the female (GENBANK SUB7440720) and the *A. alchata* male (SUB7440720) was found, confirming their conspecificity. However, this result raises a difficult issue, as Warncke believed the males of *A. d. agadira* to be identical to other *A. doursana* subspecies. This therefore means that either *A. alchata* and *A. d. agadira* are not synonymous, and simply very similar morphologically in the female sex, or the undescribed *A. d. agadira* males are incorrectly associated with the type series females. Given this uncertainty, it is not appropriate to propose synonymy between *A. d. agadira* and *A. alchata* until genetic sequences can be obtained from the *locus typicus* to clarify sex associations in this region. What is clear is that captured females represent *A. alchata*, but given the large degree of variation in the colour of *Truncandrena* pubescence, it is possible that they simply resemble females of *A. d. agadira*. As such, we do not describe females here until this situation can be clarified further.

### Other species newly recorded for the Moroccan fauna

#### Andrena (Aciandrena) pratincola

Taxon classificationAnimaliaHymenopteraAndrenidae

Warncke, 1974

FFD85076-ED79-5DE6-AF86-9338DF00E6CB

##### Material examined.

Morocco: Guelmim-Oued Noun, 10 km E Guelmim, 15–16.iv.1995, 5♂, leg. Ma. Halada, OÖLM; Drâa-Tafilalet, 20 km W Boudnib, 9.iv.1995, 1♂, leg. Ma. Halada, OÖLM.

##### Distribution and remarks.

Previously recorded from Egypt and Tunisia (Gusenleitner and Schwarz 2002), this species has red tergites in the female sex but the male is fairly generic within the *Aciandrena*; it has an almost completely yellow and polished clypeus with loose white hair (Fig. 127), the tergites are dull, the nervulus is pronouncedly antefurcal, and the genitalia are simple (Fig. 128, also illustrated in Warncke 1974). Material from Morocco was entirely male and so comparison with the female type was not possible, but all other characters in the male sex were a good match (Figs 115, 126). These records considerably extend the range of *A. pratincola* to the west. The species may be found in the Algerian part of the Sahara but more survey effort is required.

##### Other material examined.

Egypt: Min. Agr. Egypt, Dekhela, 20.ii.1917, 1♂, leg. Storey, OÖLM (paratype, illustrated Fig. 121); Ikingi, Mariout, 18.iii.1935, 1♀, leg. W. Wittmer, OÖLM (holotype); Matruh, 21.iii.1933, 1♂, leg. H. Priesner, OÖLM (paratype, illustrated Fig. 122).

#### Andrena (Aciandrena) varicornis

Taxon classificationAnimaliaHymenopteraAndrenidae

Pérez, 1895

2A6B1039-EF13-5D7F-B3AF-D5C743568959

##### Material examined.

Morocco: Oriental, 70 km S Oujda, 8.iv.1995, 1♂, leg. Ma. Halada, OÖLM.

##### Distribution and remarks.

Known from Algeria, Tunisia, Egypt, and Israel (Gusenleitner and Schwarz 2002; Pisanty et al. 2018), so the presence of this species in eastern Morocco is not unexpected. The male is unique within the *Aciandrena* in that the yellow markings on the clypeus extend on to the lower paraocular areas (Fig. 129), and additionally the tips of the gonostyli are produced into points (Fig. 130).

**Figures 125–130.**
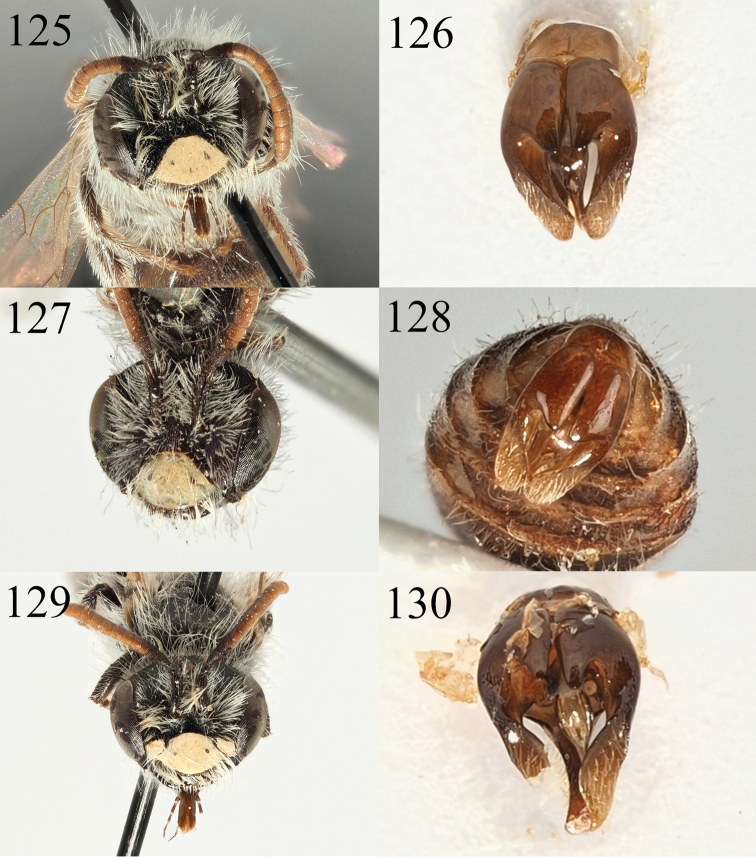
Andrena (Aciandrena) pratincola Warncke, 1974 (Morocco) **125** male face **126** male genitalia (Egypt, Paratype) **127** male face **128** male genitalia Andrena (Aciandrena) varicornis Pérez, 1895 **129** male face **130** male genitalia.

#### Andrena (Andrena) synadelpha

Taxon classificationAnimaliaHymenopteraAndrenidae

Perkins, 1914

79484202-C083-586E-9729-A45E38C12D9C

##### Material examined.

Morocco: Fès-Meknès, Ifrane environs, 1700 m, 10.v.1997, 1♀, leg. P. Průdek; South of Azrou, 25.iv.2017, 1♂, M. Snižek, both OÖLM.

##### Distribution and remarks.

This is a predominantly central and northern European bee, but there are isolated southern populations in upland areas of Spain, Portugal, and Turkey (Gusenleitner and Schwarz 2002; Wood et al. 2020a). *Andrena synadelpha* is polylectic, but it prefers pollen from trees and shrubs of broadleaf woodland such as *Acer*, *Rubus*, *Crataegus*, *Quercus*, *Rhamnus*, *Frangula* and *Ilex* (Wood and Roberts 2017). Its presence in the Middle Atlas is therefore unexpected, but it has precedence. *Andrena haemorrhoa* (Fabricius, 1781), another predominantly central and northern European bee which is able to live as far north as the Arctic circle, was also recently found in the Ouled Nail mountains of Algeria at an elevation of 761 meters (Cherair et al. 2013). It remains to be seen if there are more typically ‘European’ *Andrena* species that persist in the mountains of North Africa, presumably having been isolated at the end of the Ice Age as the lowland areas of North Africa returned to a more arid climate.

#### Andrena (Avandrena) melacana

Taxon classificationAnimaliaHymenopteraAndrenidae

Warncke, 1967

1E3DDF7E-CD84-5814-8091-B4E3B4403DB0

Replacement name for Andrena melaleuca Friese, 1922 nec Andrena melaleuca Pérez, 1895

##### Material examined.

Morocco: Fès-Meknès, 12 km east of Ifrane, 9.v.1997, 1♀, leg. J. Halada, OÖLM; Fès-Meknès, Laanoucer, 1417 m, 3.v.2018, 1♂, leg. A. Sentil & P. Lhomme, UMONS.

##### Distribution and remarks.

**Figures 131–136.**
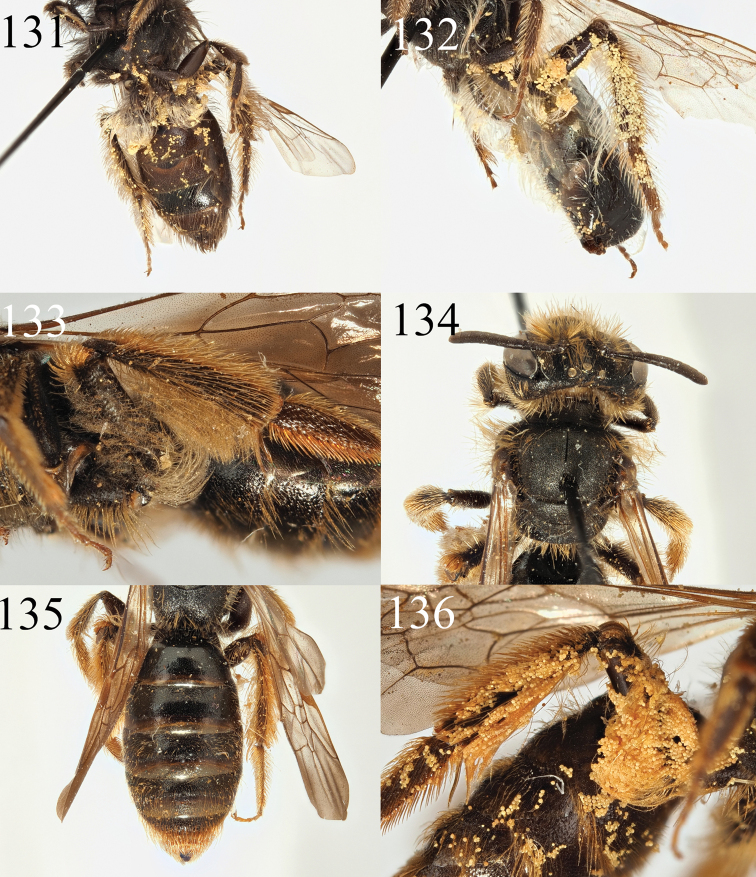
Andrena (Avandrena) melacana Warncke, 1967 **131** female scopa **132** female scopa. Andrena (Pallandrena) byrsicola Schmiedeknecht, 1900 **133** female scopa **134** female scutum **135** female tergites **136** female scopa with pollen.

Previously known from Algeria, Tunisia, and Libya (Gusenleitner and Schwarz 2002). This species is part of the *Avandrena* group that, like the *Pallandrena*, appear to be associated with Geraniaceae. Records of *A. avara* Warncke, 1967 and *A. panurgina* Desteffani, 1889 in Portugal have been made exclusively from *Erodium*, and pollen removed from the scopa from *A. melacana* comprised entirely of *Erodium*-type pollen (Figs 131, 132). *Andrena melacana* can be easily distinguished from *A. avara* and *A. panurgina* as the hind femora lacks spines (Warncke 1980) and the male genitalia are completely different.

#### Andrena (Carandrena) leucophaea

Taxon classificationAnimaliaHymenopteraAndrenidae

Lepeletier, 1841

2913DAFF-373E-5EBF-8202-6BBD7CFC3957

##### Material examined.

Morocco: Fès-Meknès, Azrou, 20 km south, 23.iv.2009, 1♀, E. & P. Hajdaj, OÖLM.

##### Distribution and remarks.

A West Mediterranean species, found in Algeria, Italy, Spain, and Tunisia (Gusenleitner and Schwarz 2002), and recently Portugal (Wood et al. 2020a). In Portugal, the bee has been recorded between January and March, and so it may have been missed by previous collectors in lowland areas of Morocco.

#### Andrena (Cryptandrena) rotundata

Taxon classificationAnimaliaHymenopteraAndrenidae

Pérez, 1895

7C4687BB-08AE-5519-8E5F-1430F012F959

##### Material examined.

Morocco: Fès-Meknès, Bhalil, 10 km NW Sefrou, 28.v.1995, 1♀, leg. Ma. Halada; Fès-Meknès, El-Menzel, 30 km E Sefrou, 29.v.1995, 2♀, leg. Mi. Halada, all OÖLM.

##### Distribution and remarks.

Known from Algeria, Italy (Sardinia), and Tunisia (Gusenleitner and Schwarz 2002). This species is very similar to *A. ventricosa* Dours, 1873 but differs by the less dense scutal punctures. Given the very slight differences between *A. rotundata*, *A. ventricosa*, and the more eastern *A. brumanensis* Friese, 1899, this group could benefit from molecular examination.

#### Andrena (Distandrena) merimna

Taxon classificationAnimaliaHymenopteraAndrenidae

Saunders, 1908

5797C30C-E2D3-50B3-B346-8B85BC3AF632

##### Material examined.

Morocco: Drâa-Tafilalet, Er-Rich, 1253 meters, 28.ii.2019, 3♂, 2♀, *Moricandia foleyi*, leg. O. Ihsane; Fès-Meknès, Laanoucer, 1387 meters, 11.v.2018, 1♀, leg. P. Lhomme & O. Ihsane, all UMONS.

##### Distribution and remarks.

Previously known only from Algeria and Tunisia (Gusenleitner and Schwarz 2002). At 9 mm in length, this one of the larger *Distandrena* species, almost as large as *Andrena fria* Warncke, 1975 which is restricted to Spain. Female Moroccan material is very slightly more striate centrally on the clypeus than comparative material from Algeria and Tunisia, but the male genitalia are a clear match.

##### Other material examined.

Algeria: Biskra, 3.ii.1997, 1♀, OÖLM; Tunisia: 30 km N Foum Tatahouine, 15.ii.1992, 1♀, leg. Warncke, OÖLM; 21.ii.1992, 1♂, leg. Warncke, OÖLM.

#### Andrena (Margandrena) menahemella

Taxon classificationAnimaliaHymenopteraAndrenidae

Scheuchl & Pisanty, 2016

B460894D-9350-52B3-B2BF-4DC2300B3066

##### Material examined.

Morocco: Fès-Meknès, south of Azrou, 25.iv.2017, 1♀, leg. M. Snižek, OÖLM; Fès-Meknès, Laanoucer, 1416 m, 11–12.iv.2019, 1♀, white pan trap; 1♀, yellow pan trap, both leg. L. Hamroud & A. Sentil, UMONS; Fès-Meknès, Ain Leuh, Azrou S, 17.iii.1990, 6♀, leg. H. Teunissen, NMNL, Leiden; Fès-Meknès, Col du Zad, 1800 m, 4.iii.1989, 1♀, 2100 m, 10.iii.1989, 2♀, all leg. H. Teunissen, NMNL, Leiden.

##### Distribution and remarks.

**Figures 137–144.**
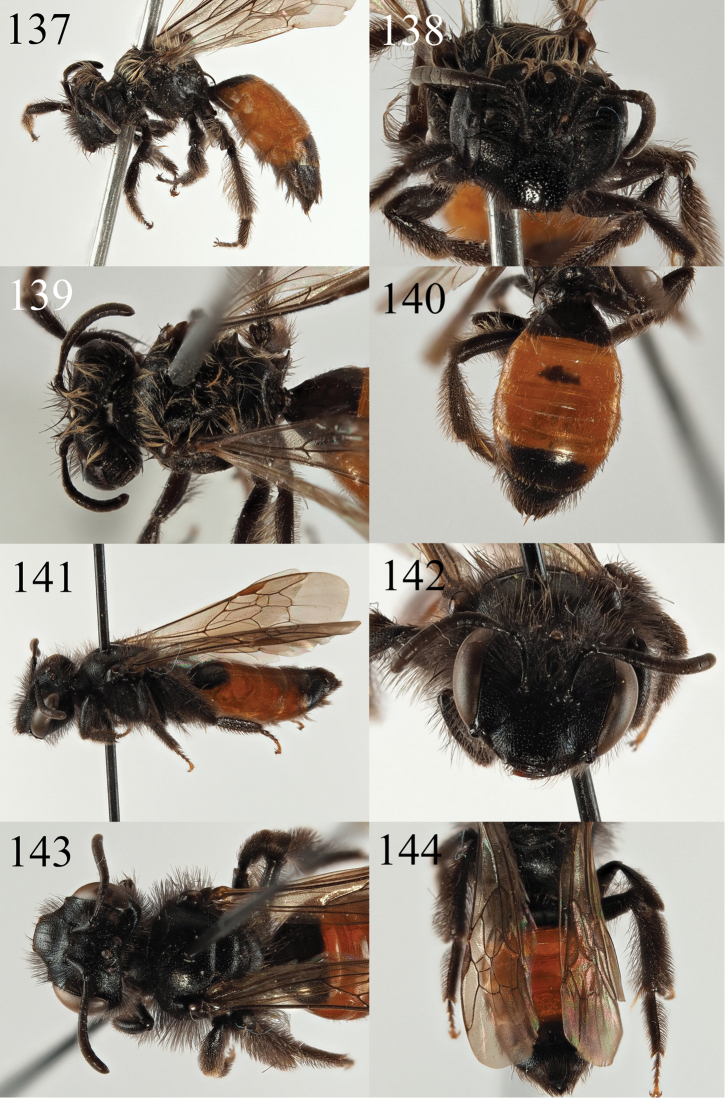
Andrena (Margandrena) menahemella Scheuchl & Pisanty, 2016 (Israel) **137** female profile **138** female face **139** female dorsum **140** female tergites (Morocco) **141** female profile **142** female face **143** female dorsum **144** female tergites.

Previously known only from central Israel (Pisanty et al. 2016, Figs 141–144). Specimens were collected at altitude from the Middle Atlas, and were also found in collections at Linz and Leiden. Specimens collected from Morocco conform to the description of *A. menahemella* with the following differences: In addition to T2+3 and S2+3, T4 is basally red and S4 is almost entirely red. There are no central black spots in T2 or T3 (compare Figs 140, 144). The outer side of the hind tibia has uniformly dark hairs (compare Figs 137, 141), and the nervulus slightly antefurcal. The hairs on the thorax are generally darker (Figs 142, 143), rather than yellowish (Figs 138, 139), contributing to an overall darker appearance.

Whilst overall the Moroccan material has more extensive reddish tergal margins and darker pubescence, the lack of any major structural differences mean that we consider this material to be conspecific with that from Israel, despite the large degree of geographic separation. This record extends the range of *A. menahemella* some 3,600 km to the west, giving a disjunct distribution of Morocco and Israel with no records from Algeria, Tunisia, Libya, or Egypt. However, this situation is not unprecedented amongst *Andrena*, with *Andrena aegyptiaca* Friese, 1899 showing a disjunct distribution being absent from much of the central part of North Africa (Gusenleitner and Schwarz 2002).

##### Floral preferences.

As the two known specimens were caught in pan traps, no information on floral preferences is available. This reflects the situation in Israel, where females are known only from pan traps (Pisanty et al. 2016). The pollen preferences of *Margandrena* are incompletely known (Standfuss and Standfuss 2010; Gogala 2011). For species that fly in the winter through to the spring, they seem to be associated with monocotyledons such as *Bellevalia* (Asparagaceae), *Colchium* (Colchicaceae), and *Crocus* (Iridaceae) (Mavromoustakis 1952, 1958; Scheuchl and Gusenleitner 2009). However, as very little detailed pollen work has been conducted on this group it is difficult to come to firm conclusions. *Andrena menahemella* should be searched for on monocotyledons in northern Morocco during March and April.

##### Other material examined.

Israel: Netiv Italamed He, 16.ii.2010, 1♀, leg. G. Pisanty, OÖLM (paratype, illustrated Figs 141–144).

#### Andrena (Micrandrena) icterina

Taxon classificationAnimaliaHymenopteraAndrenidae

Warncke, 1974

DA5F8387-C9E5-5DAC-AA12-09C7DD46DC90

##### Material examined.

Morocco: Fès-Meknès, Laanoucer, 1474 meters, 2–3.v.2018, 1♀, yellow pan trap; 33.6234, -4.9002, 11.v.2018, 2♀, all leg. P. Lhomme & O. Ihsane, UMONS.

##### Distribution and remarks.

Described from Algeria (Warncke 1974) and is also known from southern Spain (Dardon et al. 2014). Warncke originally compared *A. icterina* to the central European *Andrena strohmella* Stöckhert, 1928 and suggested an association between the two species. In a revision of *Micrandrena* species from Iberia, Dardon et al. (2014) redescribed the type of *A. icterina* noting that the distinctive carina of *A. strohmella* found on the sides of tergite 1 is absent. However, in our material the carina is present, but weak. On examination of the type material we found that the carina is present, but equally weak when compared to *A. strohmella*.

##### Other material examined.

Algeria: Teniet, 10.v.1895, 1♀, OÖLM (holotype).

#### Andrena (Micrandrena) saxonica

Taxon classificationAnimaliaHymenopteraAndrenidae

Stoeckhert, 1935

2CCD6B47-6AF2-51D9-95C4-8C3B1210742E

##### Material examined.

Morocco: Tangier-Tétouan-Al Hoceima, Issaguen, 150 km SE Tanger, 1550 m, 12.v.2015, 1♀, leg. Mucska, OÖLM; Fès-Meknès, Ifrane environs, 1700 m, 10.v.1997, 1♀, leg. P. Průdek, OÖLM.

##### Distribution and remarks.

Known from central Europe south into Spain and Greece (Gusenleitner and Schwarz 2002), and recently southern Spain (Dardon et al. 2014) and Portugal (Wood et al. 2020a). This bee is a specialist of *Ornithogalum* (Westrich 2010) and has been overlooked in southern Iberia where it is generally associated with wooded upland areas.

#### Andrena (Orandrena) monilia

Taxon classificationAnimaliaHymenopteraAndrenidae

Warncke, 1967

0B8DA960-BA71-554C-A030-D89AA928A487

##### Material examined.

Morocco: Souss-Massa, 10 km W Tiznit, 6.v.1995, 2♀, leg. Mi. Halada, OÖKM; Fès-Meknès, Laanoucer, 33.6302, -4.8847, 2–3.v.2018, 1♂; 10–11.v.2018, 2♀; 15–16.v.2018, 2♀, all caught in yellow pan traps, leg. P. Lhomme & O. Ihsane; Fès-Meknès, Laanoucer, 33.6150, -4.7752, 11–12.iv.2019, 1♀, white pan trap, leg. L. Hamroud & A. Sentil, UMONS.

##### Distribution and remarks.

Originally described from central Spain (Warncke 1967) but found also in Tunisia (Warncke 1980) and the Near East (Warncke 1969, 1976), the species was recently reported from Algeria (Benarfa et al. 2013). The presence of this species in Morocco continues to fill in the distributional gap across North Africa between Spain and the Near East.

##### Other material examined.

Spain: Montarco, 10.v.1933, leg. Dusmet, 1♀, OÖLM (holotype).

#### Andrena (Pallandrena) byrsicola

Taxon classificationAnimaliaHymenopteraAndrenidae

Schmiedeknecht, 1900

175C1181-81DA-5C38-AFF2-DD6F32D031C7

##### Material examined.

Morocco: Fès-Meknès, 12 km east of Ifrane, 9.v.1997, 1♂, 20♀, leg. J. Halada; Fès-Meknès, Ifrane environs, 9.v.1997, 2♂, 18♀, leg. K. Deneš; Fès-Meknès, Tissa environs, 8.v.1997, 1♀, leg. K. Deneš, all OÖLM.

##### Distribution and remarks.

This taxon has been poorly recorded and documented, leading to nomenclatural confusion. It is part of the Pallandrena subgenus that is characterised by females with plumose scopa on the ventral side of the tibiae (Fig. 133), smooth hind femora (contrast *Chlorandrena*), and a deeply incised labrum.

The bee is similar to *Andrena braunsiana* Friese, 1887 which is found in central Europe eastwards to Greece, Turkey and the Caucasus (Gusenleitner and Schwarz 2002). However, both the scutum (Fig. 134) and the tergites (Fig. 135) are much less strongly punctured. In Schmiedeknecht’s original description he wrote ‘Abdomen nitidum, sparsim et subtilier punctlatum, depressionibus latis apicalibus fere laevibus’ which corresponds very well to this material. In German, Schmiedeknecht draws parallels with members of the *Chlorandrena* in general impression, but notes the differences including the wide reddish margins of the tergites.

Confusion exists over this taxon because the location of the type of Schmiedeknecht collected from Tunisia is unclear and it may be lost (Gusenleitner and Schwarz 2002), and furthermore Warncke (1967) described *Andrena oblita* Warncke, 1967 from southern Italy (females, including holotype) and Tunisia (males). Grünwaldt (unpublished manuscript) examined the type series of *A. oblita* and compared it to material including a male of *A. byrsicola* collected by Schmiedeknecht from Tunis (non-type material). He found that Tunisian *A. oblita* males were identical to *A. byrsicola*, but that Italian females were identical to *A. braunsiana* females. At this moment, it is not possible to confirm these observations and to propose a formal synonymy, but both male and female Moroccan material is consistent with Schmiedeknecht’s description of *A. byrsicola*, and this taxon is likely restricted to Morocco, Algeria, and Tunisia (Kuhlmann et al. 2020).

##### Floral preferences.

There are no flower records associated with these specimens, but the unusual modified tibial scopa of the *Pallandrena* suggests some kind of floral specialisation. None of the specimens had full pollen loads, but fragments could be removed from four females (Fig. 136). Some samples were contaminated with *Cistus*-type pollen, but the dominant pollen was Geraniaceae, probably *Erodium* (84.5%). Visual inspection of the scopa of specimens of A. (Pallandrena) pallidicincta Brullé, 1832 from Greece showed the presence of similar large Geraniaceae-type grains, and *A. pallidicincta* and A. (Pallandrena) christineae Dubitzky, 2006 from Lebanon are associated with *Geranium* species (Wood et al. 2020b). It is likely that this *Andrena* clade are specialists of Geraniaceae, but more evidence is required.

#### Andrena (Parandrenella) tebessana

Taxon classificationAnimaliaHymenopteraAndrenidae

Scheuchl, Benarfa & Louadi, 2011

ACA20749-7A13-5755-ADD8-A36F99CD32BB

##### Material examined.

Morocco: Souss-Massa, 30 km north of Tafraoute (Tafraut), 7.v.1995, 1♀, leg. Ma. Halada, OÖLM; Béni Mellal-Khénifra, Khenifra env., 11.v.1997, 1♀, leg. K. Deneš, OÖLM; Casablanca-Settat, Oueled Sghir, 23.ii.2018, 8♂, 11♀; 27.iii.2018, 18♀; 4–5.iv.2018, 1♀, yellow pan trap, all leg. A. Sentil & El Abdouni, UMONS; Casablanca-Settat, Mzamza, Janoubia, 32.9524, -7.5142, 2♀, 6.v.2019, *Sinapis arvensis*, leg. A. Sentil, UMONS; Fès-Meknès, Sidi Youssef Ben Ahmed, 29.iii.2018, 1♀, leg. P. Lhomme & O. Ihsane, UMONS; Fès-Meknès, Laanoucer, 33.6150, -4.7752, 25.iv.2019, 1♀, leg. L. Hamroud & P. Lhomme, UMONS.

##### Distribution and remarks.

This species was previously known only from a small region of Tunisia and eastern Algeria (Scheuchl et al. 2011). The discovery of this species in northern Morocco extends the range of this species approximately 1,500 km to the west. It is surprising that it has not previously been detected in Morocco because these records come from four quite geographically different provinces.

##### Other material examined.

Algeria: Tébessa, Ouenza, 24.iii.2009, 1♀, OÖLM (holotype).

#### Andrena (Poliandrena) melaleuca

Taxon classificationAnimaliaHymenopteraAndrenidae

Pérez, 1895

951A66A0-778C-5514-8E2C-185DF72416DB

##### Material examined.

Morocco: Fès-Meknès, Aghbalou, Akourar, 9.v.2019, 1♀, *Marrubium vulgare*, leg. L. Hamroud & A. Sentil, UMONS.

##### Distribution and remarks.

Known from Algeria, Tunisia, and Libya (Gusenleitner and Schwarz 2002), this bee is similar to *A. macroptera* Warncke, 1974 and *A. corax* Warncke, 1967 but has a slimmer body shape than the former and has hairbands on the metasoma in contrast to the latter, as well as more subtle morphological differences in the structure of the integument.

#### Andrena (Poliandrena) relata

Taxon classificationAnimaliaHymenopteraAndrenidae

Warncke, 1967

4E478878-A512-5989-8BCC-879394347BBF

##### Material examined.

Morocco: Oriental, 40 km south of Guercif, 15–17.v.1995, 100♀, leg. Ma. Halada, OÖLM; Fès-Meknès, Ifkern, 25 km E Boulemane, 24.v.1995, 2♀, leg. Ma. Halada, OÖLM

##### Distribution and remarks.

Previously known only from Spain (Gusenleitner and Schwarz 2002), a very long series of this species was collected in eastern Morocco. Though not previously recorded from North Africa, this material matches the holotype perfectly. In Iberia, *A. relata* is known from eastern and southern Spain including from the provinces of Almería and Murcia which are the driest and most desert-like habitat in Europe, so its presence in the similarly desertic regions south of Guercif and east of Boulemane is ecologically consistent.

##### Other material examined.

Spain: Aragon, Albarracín, 5.vi.1925, 1♀, OÖLM (***holotype***); Albacete, Almansa, 25.v.1983, 1♀, leg. H. Teunissen, NMNL; Granada, Pantano de Cubillas, 27.v.1982, 1♀, leg. R. Leys, NMNL; Granada, Pantano de los Bermejales, 26.v.1982, 1♀, leg. R. Leys, NMNL; Almería, Sierra de Maria, 25 km W Lorca, 10.v.2003, 1♂, 6♀, leg. J. Halada, OÖLM; Granada, S-Sierra Nevada, env. Lanjaron, 4.v.2003, 3♀, leg. J. Halada, OÖLM; Zaragoza, Vera de Moncayo, 15.v.1995, 1♂, 1♀, leg. H. & J.E. Wiering, NMNL.

#### Andrena (Simandrena) selena

Taxon classificationAnimaliaHymenopteraAndrenidae

Gusenleitner, 1994

DC19C135-FA08-57D2-83B4-5894FF8A646B

##### Material examined.

Morocco: Souss-Massa, 20 km north Foum-Zguid, 29–30.iv.1995, 1♀, leg. Ma. Halada, OÖLM; Drâa-Tafilalet, 5 km south of Zagora, 25.iv.1995, 1♀, leg. Ma. Halada, OÖLM.

##### Distribution and remarks.

Described from desert regions of eastern Algeria, Tunisia, and Egypt (Sinai, Gusenleitner 1994), these records substantially extend the range of this species to the west in the same manner as for *A. tebessana*.

#### Andrena (Ulandrena) speciosa

Taxon classificationAnimaliaHymenopteraAndrenidae

Friese, 1899

418BBD65-9EEA-56D1-B87E-E6857C537A40

##### Material examined.

Morocco: Fès-Meknès, 20 km north of Missour, 14.v.1995, 1♀, leg. Ma. Halada, OÖLM.

##### Distribution and remarks.

Previously known from Syria, Jordan, and Israel through North Africa to Algeria (Gusenleitner and Schwarz 2002). There are only two *Ulandrena* in north-western Africa, and *A. speciosa* is instantly separable in the female sex from A. (Ulandrena) tadorna Warncke, 1974 as the former has a yellow clypeus and lower paraocular areas, whereas those of the latter are completely black.

### Species removed from the Moroccan fauna

#### Andrena (Melandrena) nitida

Taxon classificationAnimaliaHymenopteraAndrenidae

(Müller, 1776)

24AF7D61-C93A-50AF-A5C3-68974BEBA076

##### Discussion.

*Andrena nitida* was recorded from Morocco by Warncke (1974) under the name *Andrena nitida mixtura* Warncke, 1967. This subspecies was described from Portugal and Spain under the name *Andrena limata* Smith, 1853 *mixtura* (Warncke, 1967). However, Warncke transferred this subspecies into combination with *A. nitida* (Warncke 1974, 1976) without any apparent justification. Examination of the type series of *A. n. mixtura* shows that its original placement was justified based on its bivoltine behaviour and the darker colouration of its tergal and scopal hairs (Wood et al. 2020a), and that therefore records of *A. n. mixtura* from North Africa (none of which are part of the type series) refer to *A. limata* and that *A. nitida* should be removed from the Moroccan list.

##### Material examined.

Warncke Collection, OÖLM (*Andrena nitida mixtura*): Morocco: Ifrane, 18.vii.1931, 1♀, leg. A. Nadig; Koudia, 19.iii.1969, 1♀, leg. J.N. Tasei; Portugal: Carcavelhos, 29.iv.1956, 1♀, leg. N.F. d’Andrade (holotype); Coimbra, Ponte da Portela, 30.iii.1968, 1♂, leg. M.A. Diniz; Spain: Catalonia, Arenys, 15.iv.1929, 1♀, leg. Zariquiey (paratype); Playa de Aro, Gerona, 1♀, leg. H. Pochon (paratype); Catalonia, Beceite, 16.vii.1923, 1♀, leg. Zariquiey (paratype); Alicante, Orihuela, 30.v.1925, 1♀, leg. Andréu (paratype); 8.iv.1925, 1♀, leg. Andréu; 16.vi.1949, 1♂, leg. Andréu; (illegible), 8.vi.1912, 1♂, leg. J.M. Dusmet y Alonso; Barcelona, 7.vii.1898, 1♂; Tunisia: 2 km E Menzel Bourguiba, 28.iii.1976, 1♀, leg. P. Robinson.

#### Andrena (Notandrena) fulvicornis

Taxon classificationAnimaliaHymenopteraAndrenidae

Schenck, 1853

B907CFDB-40BE-53F4-B615-C9448247C8FA

##### Material examined.

Morocco: Souss-Massa, 10 km S Taroudant, 12.iv.1995, 1♀, leg. Mi. Halada, OÖLM; Tangier-Tétouan-Al Hoceima, 3 km Wm Bni Hadifa, 800 m, 15.v.1995, 1♀, leg. Aßmuth, Sanetra & Schulz, OÖLM; Fès-Meknès, 5 km SE Azrou, 31.v.1995, 2♂, leg. Ma. Halada, OÖLM; Drâa-Tafilalet, Ait sais, 23–26.v.2019, 5♀, leg. O. Ihsane, Y. Bencharki, UMONS; Béni Mellal-Khénifra, Aoulou env., 17.v.1997, 1♀, leg. J. Halada, OÖLM; Fès-Meknès, Bhalil, 10 km NW Sefrou, 28.v.1995, 12♂, 24♀, leg. Ma. Halada, OÖLM; Rabat-Salé-Kénitra, Bouknadel, 28.v.2019, 4♀, leg. I. El Abdouni & P. Lhomme, UMONS; Fès-Meknès, Fes, 23.v.1930, 1♂, leg. Werner, OÖLM; Rabat-Salé-Kénitra, Haddada, 12.vi.2018, 1♀; 8.iv-3.vi.2019, 2♀, leg. I. El Abdouni, P. Lhomme & A. Sentil, UMONS; Fès-Meknès, Ifrane, 1670 m, 11.v.2015, 1♀; leg. K. Deneš, OÖLM; Rabat-Salé-Kénitra, Kenitra, 22.vi.1987, 1♂, leg. M. Schwarz, MSC; Fès-Meknès, Laanoucer, 15.v.2018, 1♂, leg. P. Lhomme & O. Ihsane, UMONS; Lot Journu, Abjelil, 8.v.1997, 1♂, leg. P. Průdek, OÖLM; Casablanca-Settat, Mzamza Janoubia, 6–31.v.2019, 12♂, 15♀, leg. A. Sentil, UMONS; Drâa-Tafilalet, Mzizl, 22.v.2019, 1♀, leg. O. Ihsane & Y. Bencharki, UMONS; Fès-Meknès, Oued Sebou, riv, near El-Menzel, 24–27.v.1999, 1♂, 30♀, leg. P. & V. Průdek, OÖLM; Casablanca-Settat, Oueled Sghir, 23.ii-20.vi.2018, 1♂, 2♀, 10.iii-29.vi.2019, 7♂, 10♀, leg. A. Sentil, I. El Abdouni & M. Chokri, UMONS; Drâa-Tafilalet, Sidi Boukil, 24.iv-22.v.2019, 1♂, 18♀, leg. O. Ihsane & Y. Bencharki, UMONS; Drâa-Tafilalet, Tabia, 24.v.2019, 3♀, O. Ihsane & Y. Bencharki, UMONS; Fès-Meknès, Tazzeka N.P., Bab-Bou-Idir env., 28.v.1999, 1♀, leg. P. Průdek, OÖLM.

##### Distribution and remarks.

*Andrena fulvicornis* was described by Schenck in the same publication as *A. nitidiuscula* (Schenck 1853). Warncke (1967) considered the two to be synonymous under the name *A. nitidiuscula nitidiuscula* (though without formal synonymy, see Schmid-Egger and Doczkal 1995), the name for European populations and as separated from *A. nitidiuscula nigellata* Pérez, 1895 found in North Africa and the Near East (Warncke 1967; see map in Gusenleitner and Schwarz 2002), and this position persisted in future publications (Schmid-Egger and Doczkal 1995).

Schmid-Egger and Doczkal (1995) resurrected the name *A. fulvicornis* as valid on the basis of morphological differences, specifically the strength of the depressed line on the anterior part of the scutum, the density and position of punctures on the scutellum, and the colour of the hind basitarsi. There are also ecological differences, as in southern Germany *A. fulvicornis* is bivoltine whereas *nitidiuscula* is univoltine and flies only in the summer. This differentiation is also supported by more recent genetic work (Benon and Praz 2016). Whilst this distinction was clarified in central Europe, it has not yet been applied across the Mediterranean where the broader concept of Warncke (1967) has been followed in the absence of revisionary work.

At Linz, all examined material in the Warncke Collection from Algeria, Egypt, Morocco, Portugal, Spain, and Tunisia (variably identified by Warncke as *A. nitidiuscula* or *A. nitidiuscula nigellata* depending on sampling location) conformed to *Andrena fulvicornissensu* Schmid-Egger and Doczkal. Overall, examination of 273 specimens of this species pair from Iberia and North Africa revealed 272 *A. fulvicornis* [Algeria (2), Egypt (1), Morocco (161), Portugal (57), Spain (16), Tunisia (35)] and a single specimen of *A. nitidiuscula* from Portugal (Wood et al. 2020a). On this basis, *A. nitidiuscula* is removed from the Moroccan list and replaced by *A. fulvicornis*.

The identity of taxa from this complex described from North Africa previously considered as *A. nitidiuscula nigellatasensu*Warncke (1967) need to be investigated, specifically *A. nigellata* [Algeria], *A. rostellata* Pérez, 1903 [Algeria], *A. rubrosignata* Saunders, 1908 [Algeria], and A. lucens var. algira Friese, 1922 [Tunisia]. Based on the North African material examined to date, it is likely that they conform to *A. fulvicornis*, but this must be confirmed by type examination. The status of other names currently in synonymy with *A. nitidiuscula* described from parts of southern Europe including Italy (*Andrena gascheti* Pérez, 1903), Spain (*Andrena divergens* Pérez, 1903), and southern France (*Andrena petroselini* Pérez, 1903, see Gusenleitner and Schwarz 2002) needs to be assessed as they could potentially refer to either *A. nitidiuscula* or *A. fulvicornis*. *Andrena franconica* [Southwestern France] Stoeckhert, 1922 and *A. petroselini* were considered by Stoeckhert (1930) to be synonymous with *A. fulvicornis*.

True *A. nitidiuscula* is almost certainly absent from North Africa, and indeed may be rare in hot areas of Mediterranean Europe, being restricted to areas with a cooler microclimate such as the coastline of northern Portugal (Wood et al. 2020a). There is likely to be broad overlap between the two taxa from southern Germany (Schmid-Egger and Doczkal 1995) to Iberia (Wood et al. 2020a) and probably other areas of southern Europe, though *A. fulvicornis* is probably rare in cooler areas such as Switzerland (Benon and Praz 2016). In northern Europe it is highly likely that only *A. nitidiuscula* is present, with no bivoltine behaviour in this species group ever observed in Britain for example (Else and Edwards 2018).

##### Other material examined.

Senckenberg, Frankfurt (*Andrena fulvicornis*): *no collection details*, 1♀ (Neotype, designated Schwenninger 2012); (*Andrena nitidiuscula*): *no collection details*, 1♀ (Lectotype, designated Schwenninger 2013); Warncke Collection, OÖLM (all conforming to *Andrena fulvicornis*): Algeria: Algiers, 2.v.1913, 1♀; Oran, 1895, 1♂, leg. Schmiedeknecht; Egypt: Kerdasa [Kirdasah], 19.v.1929, 1♂, leg. H. Priesner; Portugal: Carcavelos, 13.vi.1953, 1♀, leg. N.F. d’Andrade; Évora, 3.vii.1953, 1♀, leg. N.F. d’Andrade; Sintra, 31.v.1953, ♀, leg. N.F. d’Andrade; Spain: Aranjuez, 26.v.1912, 1♀, leg. Dusmet; Huesca, Benasque, 12.vii.1907, 1♀; Segovia, Madrona, 30.vii.1968, 1♀, leg. K. Warncke; Sierra de Arecena, Rio Odiel dei Calanas, 25.iv.1981, 1♀, M. Kühbander; Tunisia: Tunis, 1898, 1♂, leg. Schmiedeknecht; other collections (*Andrena fulvicornis*): Tunisia: 30 km N Gabes, 10.iv.1994, 33♀, leg. M. Schwarz, M. Schwarz Colln.; Zana, 6.iv.1965, 1♀, leg. R.T. Simon Thomas, NMNL, ZMA.INS.5087405

## Conclusions

This work increases the number of *Andrena* species known from Morocco by 26, a richness increase of almost 17%. The presence of species previously known only from varying combinations of Spain, Algeria, Tunisia, Libya, Egypt, and Israel, including recently described species like *A. menahemella*, *A. selena*, and *A. tebessana*, previously undescribed species, and species more typically known from Europe such as *A. synadelpha* indicates that our knowledge of North African *Andrena* remains incomplete.

Morocco has previously been identified not only as an area supporting a rich bee fauna but also as a hotspot of bee diversity and endemism (Patiny and Michez 2007; Patiny et al. 2009). However, it is likely that several of the desert species (*A. breviceps*, *A. farinosoides*, *A. semiadesus*, *A. sparsipunctata*, and *A. triangulivalvis*) will also be present in Algeria. Species found in the Souss valley and further south (*A. acutidentis*, *A. hebescens*, and *A. tenebricorpus*) are more likely to truly be endemic, as they align more closely with the distribution of Moroccan endemics like *Camptopoeum nadigi* (Warncke, 1972), *Rophites theryi* Benoist, 1930, *Panurgus acutus* Patiny, 2002, and *Panurgus minor* Warncke, 1972 which clearly have western and Atlantic distributions within Morocco itself (Patiny and Michez 2007).

**Figure 145.**
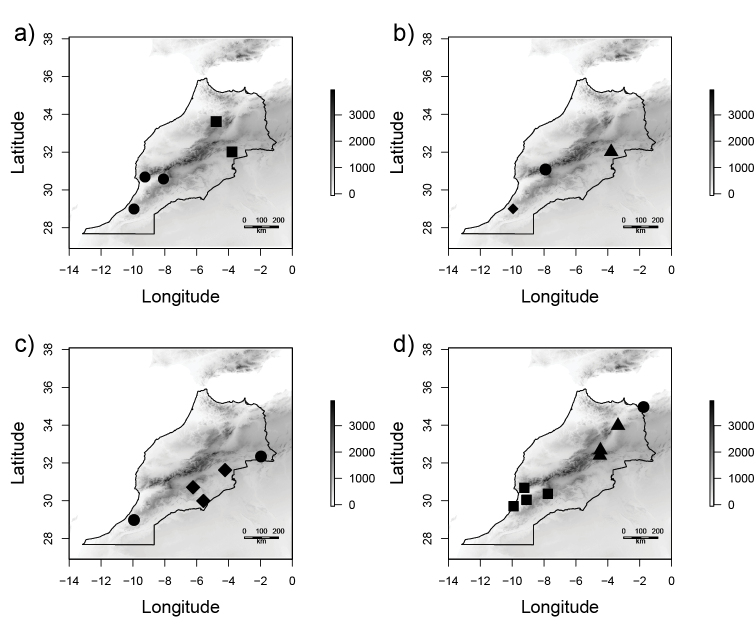
Distribution of sampling locations of newly described species **A***Andrena hebescens* (circles), *Andrena semiadesus* (squares) **B***Andrena tenebricorpus* (diamond), *Andrena niveofacies* (circle), *Andrena triangulivalvis* (triangle) **C***Andrena sparsipunctata* (circles), *Andrena breviceps* (diamonds) **D***Andrena acutidentis* (squares), *Andrena farinosoides* (triangles), *Andrena nigriclypeus* (circle). Relief is indicated by shading, measured in meters above sea level.

The distribution of sampling locations for species newly described for science (Fig. 145) shows that most were collected in desert areas, mainly in the eastern provinces of Oriental and Drâa-Tafilalet, and in the southern provinces of Guelmim-Oued Noun and Souss-Massa (the Drâa and Souss valleys). Considering the history of bee recording in Morocco, these southern and eastern areas are the most difficult to reach, and would not have been visited by historical collectors and taxonomists in the 19^th^ and early 20^th^ century. The papers of Pérez (1895), Schmiedeknecht (1900), Saunders (1908), and Alfken (1914) focused primarily on the more coastal and northern areas of North Africa that naturally were easiest to reach and therefore be visited by European collectors. Though Warncke (1974, 1980) described species collected from the Drâa and Souss valleys (e.g., *A. guichardi*), these collections were clearly not exhaustive. Other recent collecting efforts show that these desert areas are highly likely to contain further undescribed species from other bee groups; for example, a new species of *Osmia* endemic to the Souss valley was just described, (Müller 2020) and other taxonomic work has revealed a major range expansion for the poorly known genus *Borgatomelissa* (Panurginiae) that was recently found in Morocco for the first time (Ortiz-Sánchez and Patiny 2019), being previously known from the Arabian Peninsula and East Africa to Mauritania across the Sahelo-Sudanian belt.

There are many outstanding problems in North African *Andrena* arising from the large number of subspecies described or erected by Warncke such as in *A. pandosa* Warncke, 1968 and *A. medeninensis* Pérez, 1895 that require focused taxonomic attention to resolve satisfactorily, and which are beyond the scope of this paper. Dealing with these species complexes should form the base for future taxonomic research on *Andrena* in this region.

## Supplementary Material

XML Treatment for Andrena (Aciandrena) semiadesus

XML Treatment for Andrena (Aciandrena) triangulivalvis

XML Treatment for Andrena (Campylogaster) sparsipunctata

XML Treatment for Andrena (Carandrena) hebescens

XML Treatment for Andrena (Cnemidandrena) niveofacies

XML Treatment for Andrena

XML Treatment for Andrena

XML Treatment for Andrena (Notandrena) acutidentis

XML Treatment for Andrena (Poecilandrena) nigriclypeus

XML Treatment for Andrena (Poliandrena) breviceps

XML Treatment for Andrena (Poliandrena) farinosoides

XML Treatment for Andrena (Aciandrena) fulica

XML Treatment for Andrena (Nobandrena) ounifa

XML Treatment for Andrena (Poliandrena) guichardi

XML Treatment for Andrena (Truncandrena) alchata

XML Treatment for Andrena (Aciandrena) pratincola

XML Treatment for Andrena (Aciandrena) varicornis

XML Treatment for Andrena (Andrena) synadelpha

XML Treatment for Andrena (Avandrena) melacana

XML Treatment for Andrena (Carandrena) leucophaea

XML Treatment for Andrena (Cryptandrena) rotundata

XML Treatment for Andrena (Distandrena) merimna

XML Treatment for Andrena (Margandrena) menahemella

XML Treatment for Andrena (Micrandrena) icterina

XML Treatment for Andrena (Micrandrena) saxonica

XML Treatment for Andrena (Orandrena) monilia

XML Treatment for Andrena (Pallandrena) byrsicola

XML Treatment for Andrena (Parandrenella) tebessana

XML Treatment for Andrena (Poliandrena) melaleuca

XML Treatment for Andrena (Poliandrena) relata

XML Treatment for Andrena (Simandrena) selena

XML Treatment for Andrena (Ulandrena) speciosa

XML Treatment for Andrena (Melandrena) nitida

XML Treatment for Andrena (Notandrena) fulvicornis
